# Universal test, treat, and keep: improving ART retention is key in cost-effective HIV control in Uganda

**DOI:** 10.1186/s12879-017-2420-y

**Published:** 2017-05-03

**Authors:** Nicky McCreesh, Ioannis Andrianakis, Rebecca N. Nsubuga, Mark Strong, Ian Vernon, Trevelyan J. McKinley, Jeremy E. Oakley, Michael Goldstein, Richard Hayes, Richard G. White

**Affiliations:** 1 London School of Hygiene and Tropical Medicine, Keppel Street, London, WC1E 7HT UK; 2 MRC/UVRI Research Unit on AIDS, P.O. Box 49, Entebbe, Uganda; 3 School of Health and Related Research, The University of Sheffield, 30 Regent Street, Sheffield, S1 4DA UK; 4 Department of Mathematical Sciences, Durham University, Lower Mountjoy, Stockton Road, Durham, DH1 3LE UK; 5 College of Engineering, Mathematics and Physical Sciences, University of Exeter, Campusm Penryn, Penryn, TR10 9FE UK; 6 School of Mathematics and Statistics, University of Sheffield, The Hicks Building, Hounsfield Road, Sheffield, S3 7RH UK

**Keywords:** HIV, ART, Uganda, Mathematical modelling, Universal test and treat, Cost-effectiveness

## Abstract

**Background:**

With ambitious new UNAIDS targets to end AIDS by 2030, and new WHO treatment guidelines, there is increased interest in the best way to scale-up ART coverage. We investigate the cost-effectiveness of various ART scale-up options in Uganda.

**Methods:**

Individual-based HIV/ART model of Uganda, calibrated using history matching. 22 ART scale-up strategies were simulated from 2016 to 2030, comprising different combinations of six single interventions (1. increased HIV testing rates, 2. no CD4 threshold for ART initiation, 3. improved ART retention, 4. increased ART restart rates, 5. improved linkage to care, 6. improved pre-ART care). The incremental net monetary benefit (NMB) of each intervention was calculated, for a wide range of different willingness/ability to pay (WTP) per DALY averted (health-service perspective, 3% discount rate).

**Results:**

For all WTP thresholds above $210, interventions including removing the CD4 threshold were likely to be most cost-effective. At a WTP of $715 (1 × per-capita-GDP) interventions to improve linkage to and retention/re-enrolment in HIV care were highly likely to be more cost-effective than interventions to increase rates of HIV testing. At higher WTP (> ~ $1690), the most cost-effective option was ‘Universal Test, Treat, and Keep’ (UTTK), which combines interventions 1–5 detailed above.

**Conclusions:**

Our results support new WHO guidelines to remove the CD4 threshold for ART initiation in Uganda. With additional resources, this could be supplemented with interventions aimed at improving linkage to and/or retention in HIV care. To achieve the greatest reductions in HIV incidence, a UTTK policy should be implemented.

**Electronic supplementary material:**

The online version of this article (doi:10.1186/s12879-017-2420-y) contains supplementary material, which is available to authorized users.

## Background

Approximately 1.5 million people died from HIV-related illnesses in 2013, with sub-Saharan Africa accounting for 74% of deaths [1]. In the same year, 2.1 million people were newly infected with HIV. Uganda had an adult (15–49 years) HIV prevalence of 7.3% at the time of the last prevalence survey in 2011, and it is estimated that around 95,000 people were newly infected with the virus in 2014 [2]. Anti-retroviral therapy (ART) coverage of all HIV infected adults in Uganda was estimated to be around 51% in 2014 [2].

UNAIDS recently announced ambitious new targets to ‘end AIDS by 2030’ – fewer than 200,000 new infections among adults- with goals for 2020 of 90% of HIV positive people knowing their status, 90% ART coverage among people who know their status, and 90% viral suppression among people on ART [3]. The Ugandan Ministry of Health targets are equally ambitious: their 2015/2016–2019/2020 National HIV and AIDS Strategic Plan sets the goal of a 70% reduction in adult HIV incidence by 2020 [4]. To achieve these goals, ART coverage in Uganda will need to increase dramatically over the next few years.

ART and HIV care coverage in Uganda and other sub-Saharan African countries could be increased in a range of different ways [5–20], and it is not clear what is the most cost-effective option. Uganda’s Strategic Plan lists a number of objectives, including scaling-up coverage of HIV testing, increasing linkage to care, and strengthening community level follow-up and treatment support mechanisms for people in pre-ART and ART care [4]. Other potential options include adopting the latest WHO guidelines, which recommend ART for all people diagnosed as HIV positive [21], or adopting a ‘universal test and treat’ strategy, combining universal ART eligibility for all HIV positive people with a comprehensive programme of HIV testing [20].

In this study, we use mathematical modelling to estimate the costs and effects of different ART scale-up options, and identify the most cost-effective options at different willingness to pay (WTP) per disability-adjusted life-year (DALY) averted thresholds.

## Methods

### Model description

**Fig. 1:**
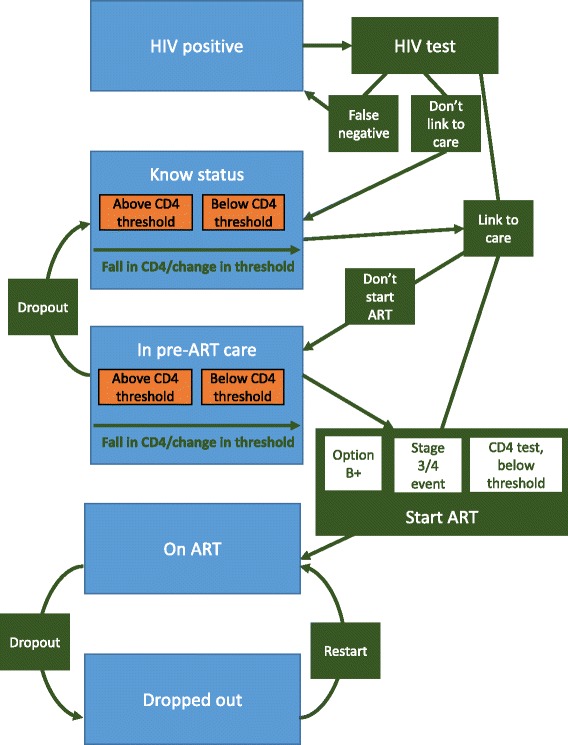
Summary of the simulated care pathway

A dynamic, agent-based model of HIV transmission and ART scale-up was developed in NetLogo [22]. The model simulates the formation and dissolution of sexual partnerships, HIV transmission, pre-ART and ART, and drug resistance. The model was designed to accurately represent major routes into and through HIV care in Uganda (summarised in Fig. 1). A full description is given in Additional file 1.

### Data sources and analysis

The model was fitted to data on demography and trends in HIV prevalence over time in Uganda; data on sexual behaviour from a rural general population cohort in South-West Uganda [23, 24]; and routinely collected national data on the proportion of HIV+ adults who were on ART, the proportion of ART initiators who started with a CD4 count of <250 cells/μl, in 2005, 2007, 2009, 2011, and 2013, and 12-month retention on ART in 2014 [25, 26]. The square root of CD4 count was assumed to decline linearly over time [27]. To capture the effects of the introduction of Option B+ in Uganda, the model was fitted to data on the proportion of ART initiators who were women in 2010, and the increase in the proportion who were women between 2010 and 2014 [25, 28].

Ethical approval for the sexual behaviour data collection and analysis was granted by the Uganda Viral Research Institute Ethics committee and the Ugandan National Council for Science and Technology. All other data used were obtained from publicly available sources.

In total, 51 outputs were fitted, and 96 inputs were allowed to vary during the fitting process. For full details see Additional file 1, which includes a table of input and output ranges; Additional file 2, which explains the rationale for the choices of input and output ranges; and Andrianakis et al. (2016) [29].

### Fitting method

The model was fitted to the empirical data using history matching with model emulation, which allowed uncertainties in model inputs and outputs to be fully represented, and allowed realistic estimates of uncertainty in model results to be obtained. History matching is a procedure that identifies and iteratively rejects parts of the model’s input space (input combinations) that are unlikely to produce model outputs within the plausible ranges [29, 30]. The model is first run at a range of different input parameter combinations, spanning the range of the model’s input space. Emulators are then trained using these model runs, and are used to predict the value of model outputs at points between the model runs. Areas of input space where the emulator predictions are very far from the empirical data are then discarded. This process is then repeated iteratively, with the model input space shrinking each time. The process is stopped when the input space is sufficiently small for an adequate proportion of model runs to fit the model outputs. Further details are given in Additional file 2, and in Andrianakis et al. (2016) [29]. Using this method, we generated 100 model fits.

### Baseline and interventions

Using the calibrated model, we explored the effects of six different intervention components:

1. Increased HIV testing (testing rate doubled)
2. No CD4 threshold for ART initiation
3. Improved retention on ART (drop-out rates halved)
4. Improved ART restart rates (restart rates doubled)
5. Improved linkage to care (linkage probability doubled)
6. Improved pre-ART care (pre-ART drop-out rates halved, rate of starting ART from pre-ART care when eligible doubled, linkage probability doubled)

Full details are given in Additional file 1. Using these six intervention components, a total of 21 intervention scenarios were simulated:

1. Each single intervention component (six interventions).
2. All plausible combinations of two components (13 interventions) (interventions combining universal access to ART and improved pre-ART care were considered implausible).
3. Two intensive, multi-component interventions: universal test and treat (UTT; components 1,2, and 5) and universal test, treat, and keep (UTTK; components 1–5).

The costs and effects of different intervention scenarios were compared to a baseline scenario, where no interventions were implemented. Interventions were implemented at the start of 2016, and the model was run until the end of 2030. Results were averaged over 2000 (stochastic) repetitions for each scenario and model fit.

For the purposes of some analyses, it was beneficial to have estimates of the costs and effects of all plausible combinations of three or more intervention components. Due to computing resource constraints, it was not possible to explicitly simulate all 21 (including UTT and UTTK) of these interventions. Instead, the costs and benefits of three/four component interventions were estimated by summing the effects of simulated interventions. Full details are given in Additional file 2.

### Costs and DALYs

A health-systems approach was used in calculating costs. Twenty cost parameters were used in calculating the costs of each scenario. Fifteen of the costs were common to all interventions, and included 1st and 2nd line drug costs; healthcare costs arising from HIV-related morbidity while not in care, in pre-ART care, and on ART (by CD4 count); programme costs for pre-ART and ART programmes; and HIV and CD4 test costs. Five costs were directly related to the costs of implementing the interventions, and included increased programme costs, decreased or increased costs of additional HIV tests, and increased costs of positive HIV tests for interventions that increased linkage to care. Plausible ranges for cost parameters were determined using published data sources. Plausible ranges and sources for cost parameters are given in Table 1, and full details are given in Additional file 2.

**Table 1:** Plausible ranges and sources of cost and DALY parameters

Name	Description	Plausible range	Source
1st_‌line_‌drug_‌cost	Annual cost of 1^st^ line antiretroviral drugs, per person	118–137 USD	Uganda Ministry of Health (2013) [31] and WHO (2015) [32]
2nd_‌line_‌drug_‌cost	Annual cost of 2^nd^ line antiretroviral drugs, per person	151–330 USD	Uganda Ministry of Health (2013) [31] and WHO (2015) [32]
preART_‌program_‌cost	Annual pre-ART program costs, per person	79–316 USD	Menzies et al. (2011) [33]
early_‌ART_‌program_‌cost	Annual program costs of providing ART for 1st six months, per person	112–449 USD	Menzies et al. (2011) [33]
reduced_‌cost_‌established_‌ART	Reduction in program costs after 6 continuous months on an ART regimen	0.7–1	Menzies et al. (2011) [33]
HIV_‌test_‌cost	Cost per HIV test	5.51–7.05 USD	Nichols et al. (2014) [34] and Mulogo et al. (2013) [35]
CD4_‌test_‌cost	Cost per CD4 test	5.18–17.48 USD	Kahn et al. (2011) [36] and Lara et al. (2012) [37]
clinic_‌visit_‌cost	Average cost per clinic visit (due to HIV-related morbidity)	2.49–9.94 USD	Pitter et al. (2007) [38]
hospital_‌night_‌cost	Average cost of a night’s stay in hospital	3.95–15.80 USD	Pitter et al. (2007) [38]
nights_‌per_‌hospital_‌visit	Average duration of an inpatient hospital stay, in nights	3–7	Pitter et al. (2007) [38]
‌hospital_‌nights_‌parameter	Determines the relationship between CD4 count and the rate of inpatient hospital stays per year for HIV+ people not receiving ART or pre-ART care (see Additional files 1 and 2 for details)	−147.9 - -79.4	Mermin et al. (2008) [39]
reduced_‌hospital_‌pre-ART_‌care	Reduction in inpatient hospital visits for HIV+ people receiving pre-ART care	0.48–0.98	Mermin et al. (2008) [39]
reduced_‌clinic_‌pre-ART_‌care	Reduction in clinic visits for HIV+ people receiving pre-ART care	0.73–0.995	Mermin et al. (2008) [39]
reduced_‌hospital_‌ART	Increased reduction in inpatient hospital visits for HIV+ people on ART compared to people receiving pre-ART care	0.32–0.78	Mermin et al. (2008) [39]
clinic_‌hospital_‌visit_‌ratio	Ratio of clinic visits to inpatient hospital stays	2–5	Mermin et al. (2004) [40]
additional_HIV_test_increased_cost	Increased cost of HIV tests conducted as part of an intervention (relative to baseline cost)	−0.5 - 0.5	Menzies et al. (2009) [41] and Tumwesigye et al. (2010) [42]
improved_linkage_to_care_cost	Increase in cost per positive HIV test associated with interventions to improve linkage to care.	0–20 USD	Expert knowledge
reduced_ART_drop_out_cost	Increase in ART program costs per person per year to improve retention	0–50 USD	Chang et al. (2010) [43] and Chang et al. (2013) [44]
increase_ART_restart_cost	Cost of increasing ART restart rates per dropout per year	0–50 USD	Chang et al. (2010) [43] and Chang et al. (2013) [44]
reduced_preART_drop_out_cost	Increase in pre-ART program costs per person per year to improve pre-ART care	0–50 USD	Chang et al. (2010) [43] and Chang et al. (2013) [44]

Full details of the cost and DALY parameters used are given in Additional file 2

Four DALY parameters were used in estimating the impact of interventions: a parameter which determined the relationship between CD4 count and disability, a disability weight for people who had continuously been on ART for more than six months, a parameter which determined the reduction in morbidity while in pre-ART care, and a parameter that determined the reduction in morbidity during the first six months on ART. Plausible ranges and sources for cost parameters are given in Table 1, and full details are given in Additional file 2.

All costs and DALYs were discounted by 3% per year. DALYs were not age-weighted. For each of the 100 model fits, 20 parameter sets were created, with different cost and DALY parameter values. This gave a total of 2000 parameter sets. Cost and DALY parameter values for the 2000 parameter sets were selected using Latin hypercube sampling. For each intervention and parameter set, the additional costs of the intervention, and the additional number of DALYs averted (in comparison to the baseline scenario) were calculated. Full details are given in Additional file 2.

The incremental net monetary benefit (NMB) of each intervention for each parameter-set was calculated, for a wide range of different willingness/ability to pay (WTP) per DALY averted thresholds (the value ascribed to a DALY) ($0–$2500), using the formula *NMB = DALYs averted x WTP – cost*. The most cost-effective intervention for each parameter-set at a WTP threshold was considered to be the intervention with the highest NMB. We present our results using both WHO thresholds (results section ‘Cost-effectiveness (WHO thresholds)), and using a net monetary benefit approach (results section ‘Net monetary benefit’).

### Sensitivity analysis

In the main analyses, all people aged 50+ years were removed from the model, and neither their costs to the health services nor DALYs averted were considered. In a sensitivity analysis, we explored the effects of including the costs and DALYs averted in people aged 50–69. Full details are given in Additional file 2.

## Results

### Fit to data

**Fig. 2:**
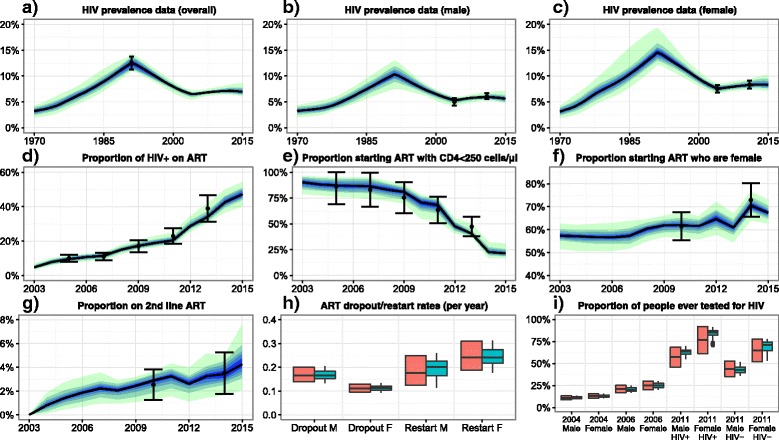
Model baseline fit to empirical data. Graphs **a**-**f**: Black dots show the empirical estimates, and the error bars show the plausible ranges for the output values. Black lines show the median model output. Blue/green bands show 10% quantiles of model outputs, from the 100 model fits. The full width of the band shows the range of the model output. Graphs **g**-**i**: Orange boxes show the empirical data and plausible ranges. Green boxes show the model output. Model fits to the remaining 20 outcomes are show in Additional file 3

**Fig. 3:**
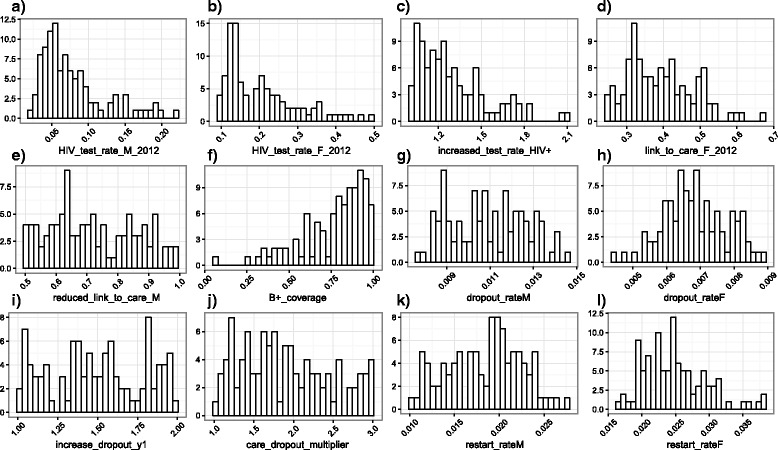
Histograms of key input parameter values in the 100 model fits. **a** Baseline* rate of testing for HIV per month from 2012, in men who have not been tested within the past 6 months. **b** Baseline* rate of testing for HIV per month from 2012, in women who have not been tested within the past 6 months. **c** Increased rate of testing in HIV+ people (multiplicative). **d** Baseline* proportion of women linked to care following a positive HIV test, from 2012. **e** Proportion of men linked to care following a positive HIV test, from 2012, relative to proportion of women. **f** Coverage of B+ (following its adoption). **g** Baseline* rate of dropping out of ART in men, per month. **h** Baseline* rate of dropping out of ART in women, per month. **i** Increased rate of dropping out of ART in the first 12 months following ART initiation. **j** Increased rate of dropping out of pre-ART care, relative to the rate of dropping out of ART. **k** Baseline* rate of restarting ART in men, per month. **l** Baseline* rate of restarting ART in women, per month. *Before adjustment for adherence/health seeking behaviour. Histograms for all model inputs are shown in Additional file 4

The model fitted closely to the plausible ranges for all outputs. Figure 2 shows the fit to data for 31 outputs, including male and female HIV prevalence over time, ART coverage (among all HIV positive people), and HIV testing. Fits to the other 20 model outputs are shown in Additional file 3. Histograms of key input parameter values in the 100 model fits are shown in Fig. 3, and for all input parameters in Additional file 4.

### Impact of interventions

#### Impact on HIV incidence in 2030

**Fig. 4:**
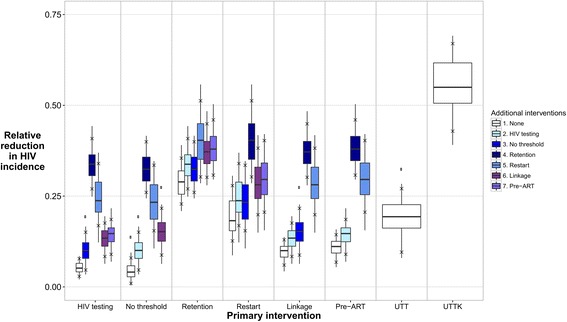
Relative reduction in HIV incidence in 2030 in the intervention scenarios, compared to baseline. Boxes show the median and 25–75% quartiles. Crosses show the 90% plausible range. White boxes show the results for the various single intervention components, UTT, and UTTK. Shaded boxes show the results for combinations of two intervention components. Results for two-component interventions are shown twice, once for each intervention component

Figure 4 shows the reduction in HIV incidence in 2030 in the intervention scenarios compared to baseline. The reductions in incidence were: 5.2% (90% plausible range of 2.9–7.8%) with increased HIV testing, 4.1% (0.97–8.8%) with no CD4 threshold, 29% (23–35%) with improved retention on ART, 18% (13–28%) with increased ART restart rates, 10% (6.0–13%) with improved linkage to care, 11% (6.8–14%) with improved pre-ART care, 19% (9.6–27%) with UTT, and 55% (43–67%) with UTTK.

#### Cost-effectiveness (WHO thresholds)

**Fig. 5:**
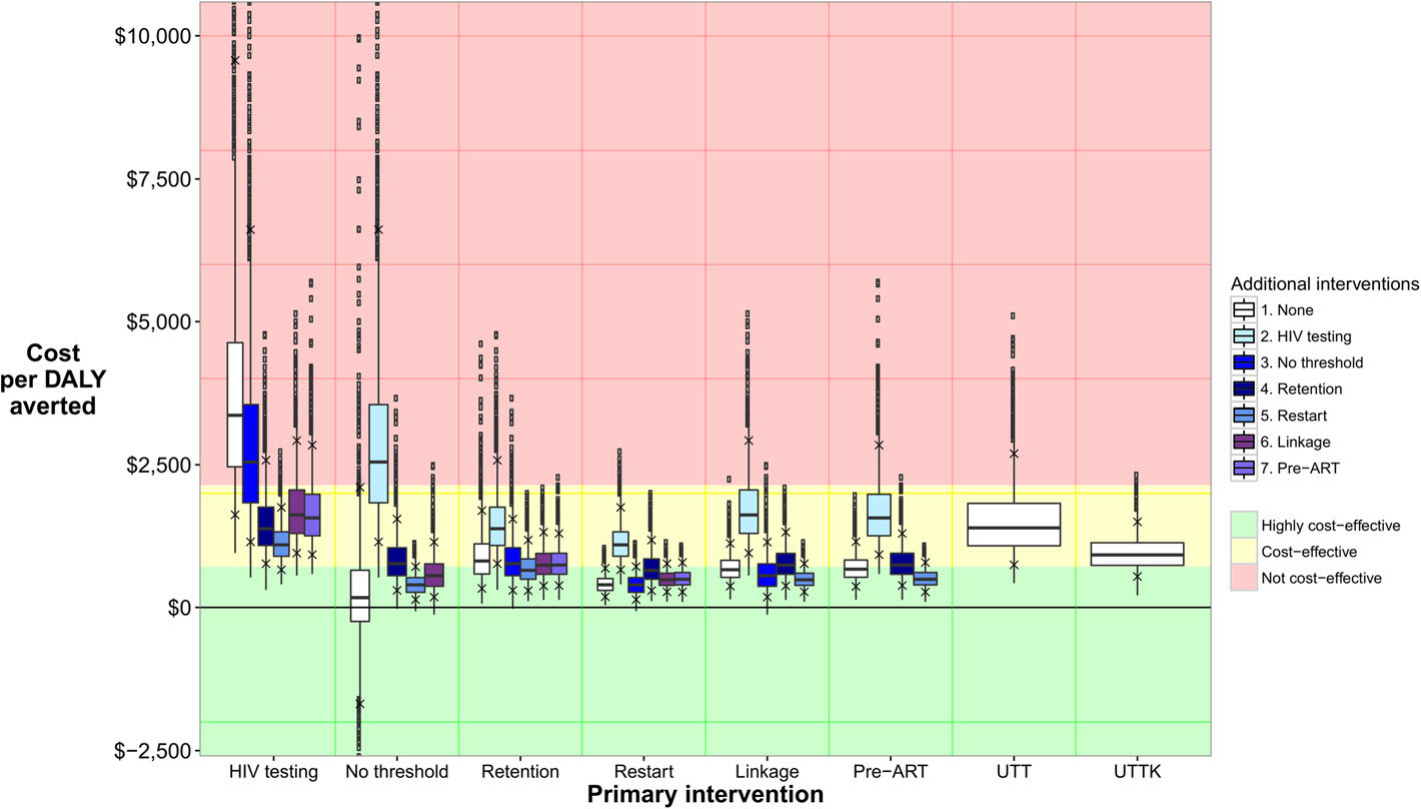
Distribution of cost per DALY averted for each intervention. White boxes show the results for single intervention components, UTT, and UTTK. Shaded boxes show the results for combinations of two intervention components. Boxes show the median and 25–75% quartiles. Crosses show the 90% plausible range. Results for two-component interventions are shown twice, once for each intervention component. Red, yellow, and green bands show areas where intervention are considered not cost-effective (cost >3 × Uganda’s per capita GDP per DALY averted, >$1430), cost-effective (cost 1–3 × Uganda’s per capita GDP per DALY averted, $715–$1430), and highly cost-effective (cost <1 × Uganda’s per capita GDP per DALY averted, <$715) respectively. In this figure, parameter sets are excluded from the results for an intervention if the number of DALYS averted is less than zero. The maximum number of parameter sets excluded for any intervention is 134/2000 (6.7%)

The majority of interventions were highly cost-effective (cost less than Uganda’s per capita GDP per DALY averted) or cost-effective (cost between one and three times Uganda’s per capita GDP per DALY averted) in more than 75% of parameter sets (Fig. 5). The two exceptions were increased HIV testing, and a combination of increased HIV testing and removing the CD4 threshold for ART initiation, which were not cost-effective (cost more than three times Uganda’s per capita GDP per DALY averted) for the majority of parameter sets. Plots of DALYs averted against intervention costs are shown in Additional file 3.

#### Net monetary benefit

**Fig. 6:**
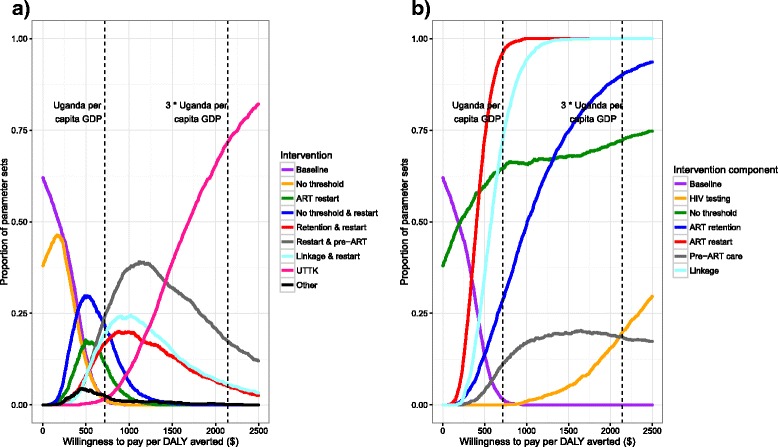
Cost-effectiveness acceptability curves. **a** Lines show the proportion of parameter sets for which an intervention is the most cost-effective option (highest net monetary benefit), for different willingness to pay per DALY averted thresholds. Interventions which are the most cost-effective option in less than 5% of scenarios at all willingness to pay thresholds are combined into the single category ‘other’. **b** Lines show the proportion of parameter sets where the most cost-effective intervention includes each individual intervention component, for different willingness to pay per DALY averted thresholds. Combinations of three and four interventions were included in the analysis for Fig. 6b, but not for Fig. 6a

Figure 6a shows a cost-effectiveness acceptability curve for all intervention options (including baseline), at willingness to pay thresholds (WTP) of $0–2500 per DALY averted. With a WTP of $0, removing the CD4 threshold is more cost-effective (has higher net monetary benefit) than the baseline scenario in 38% of parameter sets, indicating that it may be cost-saving. At intermediate thresholds of around $400–1500 (0.56–2.1 times per capita GDP), there is a large degree of uncertainty concerning the most cost-effective option. However, the model suggests that interventions that include increased HIV testing are unlikely to be among the most cost-effective options. At higher thresholds of above $1690 (2.4 times per capita GDP), UTTK is the most cost-effective option in 50–82% of parameter sets.

Figure 6b shows the proportion of parameter-sets where each individual intervention component is included in the most cost-effective intervention option, for WTP of $0–$2500. This was generated using the estimated costs and effects of both simulated interventions, and interventions combining three or more intervention components (see Additional file 2 for further details of the combined interventions). Removing the CD4 threshold is included in the most cost-effective intervention in 50–75% of parameter sets for all WTP thresholds >£210 per DALY averted. Increasing ART restart rates and improving linkage to care are included in >50% of parameter sets at WTP > $400 and WTP > $570 respectively, reaching 100% of parameter sets at WTP > $980 and WTP > $1770. The proportion of parameter-sets where the most cost-effective intervention includes improving ART retention increases gradually at WTP thresholds of >$100, reaching >50% of parameter-sets at $1010, and >75% at $1510. The proportion of parameter-sets where improved pre-ART care is included in the most cost-effective option never increases above 23%. Finally, increased rates of HIV testing is never part of the most cost-effective intervention at WTP thresholds of below $650, and is part of the most cost-effective option in a maximum of 30% of parameter sets at the maximum threshold we considered of $2500.

#### Sensitivity analysis

Including the costs and DALYs averted in people aged 50–69 had little effect on the results of any of the analyses (see Additional file 3).

## Discussion

Our model results suggest that the optimum intervention varies with the WTP per DALY averted. At all WTP > $210, removing the CD4 threshold for ART initiation is included as part of the most cost-effective intervention for more than 50% of parameter-sets. In addition, it is cost-saving for 38% of parameter sets. Based on this, we recommend that the CD4 threshold for ART initiation in Uganda is removed, in line with WHO guidelines. At intermediate WTP thresholds of around $715 (Uganda’s per capita GDP in 2014), there is large amount of uncertainty in the optimum intervention(s). Our results suggest, however, that interventions aimed at improving linkage to and retention/re-enrolment in HIV care are highly likely to be more cost-effective than interventions to increase rates of HIV testing in the general population. At these thresholds, universal access to ART for all people diagnosed with HIV should be supplemented with additional interventions aimed at improving linkage to and/or retention in HIV care. Finally, at high WTP thresholds of above around 2.4 times Uganda’s per capita GDP, we found that universal test, treat, and keep (UTTK) was the most cost-effective option.

UNAIDS recently published ambitious new targets to ‘end the AIDS epidemic by 2030’ [3]. To achieve this, it will be necessary to intensively scale-up HIV care programmes and ART provision throughout sub-Saharan Africa. A ‘universal test and treat’ policy – combining large-scale programmes of HIV testing with universal access to ART – is frequently promoted as a way of greatly reducing HIV incidence and mortality. We demonstrate that improving retention on ART should also constitute a key policy component in the drive to eliminate HIV. For this reason, we advocate a ‘universal test, treat, and keep’ policy.

A great strength of our work is the comprehensive incorporation of a large number of the potential sources of uncertainty in our results, through allowing a large number of model inputs to vary during the model fitting, and through fitting to realistic plausible ranges on a large number of model outputs. This was made possible through our use of an innovative fitting method: history matching using model emulation. In addition to this, we also incorporated uncertainty in costs and HIV disability weights in our analysis of the model output. Our results demonstrate the critical importance of this process. While we are very confident in our recommendations for low and high WTP thresholds, there is far more uncertainty in the optimum intervention at intermediate WTP thresholds of around one times Uganda’s GDP. Inadequate representation of the uncertainty that exists in current conditions would have led to an underestimate of the uncertainty in future predictions. This in turn would have led to overconfident, and potentially deleterious, recommendations being made.

We chose to model the effects of the different interventions components – for instance a 50% reduction in the rate at which people drop out of ART – rather than the activities themselves – for instance the use of peer health workers to encourage people to remain in care – to increase the generalisability of the results. Whether possible, both key resource costs (e.g. drugs and care for HIV-related morbidity), and the costs of implementing interventions, were informed by empirical studies from Uganda. There were some gaps in the empirical cost data available however, including in the additional costs required to scale-up interventions. We therefore chose wide acceptable ranges for intervention cost parameters. This meant that the uncertainties in input parameter values were accounted for in our analyses, as they were projected forward into the uncertainty estimates we show for our results, and the levels of confidence we place in our conclusions.

Our results strongly suggest that an increase in the rates of HIV testing in the general population in Uganda is only likely to be a cost-effective option at high WTP thresholds, and that it should not be prioritised above interventions to improve linkage to, and retention in, care. This reflects the fact that overall rates of HIV testing in Uganda are already relatively high: approximately 50% of Ugandan adults were tested and received their results in 2014 [2]. Interventions to increase rates of HIV testing may be more cost-effective in other populations, both in countries with lower general population rates of HIV testing, and in sub-populations in Uganda with lower testing rates and/or higher HIV incidences.

A limitation of our work is that we were unable to simulate all possible combinations of individual intervention components, due to computing resource constraints. Comparing the costs and DALYs averted of single vs double interventions suggested there was little interaction between most pairs of intervention components (Additional file 3). This allowed us to combine interventions additively for the purposes of determining if an individual intervention component is included in the most cost-effective intervention for each parameter-set and WTP. This approach may underestimate uncertainty in the differences in costs and effects between different interventions however, and so was not used for the main analysis. The single three-component intervention we simulated (UTT) was highly unlikely to be more cost-effective than UTTK at any WTP. It is nevertheless possible that three or four component interventions that we did not simulate may be the most cost-effective option at some WTP thresholds, particularly at WTP thresholds around the range where UTTK first becomes the optimum intervention.

## Conclusions

We recommend that the CD4 threshold for ART initiation in Uganda is removed, in line with current WHO guidelines. At higher WTP thresholds, and if sufficient resources are available, this should be supplemented with interventions aimed at improving linkage to and/or retention in HIV care. Finally, to achieve the greatest reductions in HIV incidence, a universal test, treat and keep policy should be implemented, combining increased rates of HIV testing, universal access to ART for all people diagnosed with ART, and measures to improve retention in care. More generally, in Uganda, interventions to improve retention in and movement through the HIV care pathway should be prioritised over case finding interventions in the general population.

## Additional files

Additional file 1: Technical model description. (DOCX 157 kb)

Additional file 2: Model and data description. (DOCX 460 kb)

Additional file 3: Supporting results. (DOCX 4161 kb)

Additional file 4: Histograms of input parameter values in the 100 model fits. (DOCX 1646 kb)

## Abbreviations

ART: Anti-retroviral therapy
DALY: Disability adjusted life year
GDP: Gross domestic product
HIV: Human immunodeficiency virus
NMB: Net monetary benefit
UNAIDS: Joint United Nations Programme on HIV/AIDS
UTT: Universal test and treat
UTTK: Universal test, treat, and keep
WHO: World Health Organization
WTP: Willingness to pay
