# Supplementary material for: Universal test, treat, and keep: improving ART retention is key in cost-effective HIV control in Uganda
Source: BMC Infect Dis. 2017 May 3;17:322. doi: 10.1186/s12879-017-2420-y (PMC5415795; doi:10.1186/s12879-017-2420-y)
Supplement: Supplementary file 1 — Technical model description. (DOCX 157 kb) [file 12879_2017_2420_MOESM1_ESM.docx]

# Technical model description

## Guide to pseudo-code

Pseudo-code is used throughout the model description to provide a clear, complete, and reproducible guide to the structure of the model. It does not include all of the code that goes into tracking and producing outputs.

Text colours and formatting are used to improve the readability of the pseudo-code. The names of model-wide parameters are written in blue. The names of attributes belonging to individual agents in the model are written in purple. Instructions or commands are written in red. The names of groups of agents are written in **bold**. Where submodels are called from within other submodels, the names of the submodels are written in orange. Where agents are telling other agents to do a task, the task required of the agent is written in *italics*. Text in green is comments, and not part of the submodel.

In many places in the model, simulated men and women are ‘asked’ to do tasks, where the only difference between the tasks is due to a single parameter. E.g. **men** may be asked to test for HIV at rate HIV_test_rate_M, and **women** may be asked to test at rate HIV_test_rate_F. To prevent unnecessary duplication in the model description, this is written as: ask **(wo)men** if random-uniform [0,1) < HIV_test_rate_M/F…

The individual attribute ART_status has seven possible categories in the model: never, 1^st^ line ≤1 year, 1^st^ line >1 year, 2^nd^ line ≤1 year, 2^nd^ line >1 year, dropped out of 1^st^ line, and dropped out of 2^nd^ line. To simplify the model description, an additional seven categories are used, consisting of combinations of the other categories:

1. on ART (1^st^ line ≤1 year, 1^st^ line >1 year, 2^nd^ line ≤1 year, or 2^nd^ line >1 year)
2. ART ≤1 year (1^st^ line ≤1 year, or 2^nd^ line ≤1 year)
3. ART >1 year (1^st^ line >1 year, or 2^nd^ line >1 year)
4. on 1^st^ line ART (1^st^ line ≤1 year, or 1^st^ line >1 year)
5. on 2^nd^ line ART (2^nd^ line ≤1 year, or 2^nd^ line >1 year)
6. not on ART (never, dropped out of 1^st^ line, or dropped out of 2^nd^ line)
7. dropped out (dropped out of 1^st^ line, or dropped out of 2^nd^ line)

## Entities and scale

There are two types of entities in the model, people and sexual partnerships.

The model has a time step of one month. For simplicity, all months are assumed to have a length of 365.25/12 days. Simulations were run for 972 months between January 1950 and December 2030. The model has no spatial scale.

## State variables

The tables of state variables give lists of all of the individual-level attributes or variables that need to be known to fully record the state that each person (Table 1) or partnership (Table 2) in the model is in.

Table 1. Human state variables

| Class | Name | Description | Type of parameter | Fixed or varying? |
| --- | --- | --- | --- | --- |
| Demographic | ID_‌number | Unique number assigned to each person at model entry | Whole number | Fixed |
|  | age | Age in months above 15 years | Rational number. | Varying. Increased over time at a rate of one year/year |
|  | sex | Sex | Binary. Male or female | Fixed |
| Behaviour | risk_‌group | Sexual behaviour risk group | Binary. High or low | Fixed |
|  | concurrency_‌group | Concurrency risk group | Binary. High or low | Fixed |
|  | adherence_‌group | Measure of health seeking behaviour and adherence to treatment | Binary. High or low | Varying. Can change from high to low on when first starting ART. Otherwise, fixed. |
| HIV related | HIV_‌status | HIV status | Binary. Negative or Positive | Varying. |
|  | primary_‌infection | Is the person in the primary stage of HIV infection | Binary. Yes or No | Varying. No on model entry, changes to yes when first infected, reverts to no after a fixed, person-specific length of time. |
|  | CD4_‌count | CD4 count. Cells/µL | Rational, positive number. | Fixed when HIV negative. Declines over time when HIV positive and not on ART. |
|  | CD4_‌decline_‌rate | Rate of decline in √CD4 per month when HIV positive and not on ART | Rational, positive number. | Fixed |
|  | primary_‌time | Time spent in primary infection stage following infection | Rational, positive number. | Fixed |
|  | time_‌since_‌infection | Number of months since HIV infection | Whole number. | Varying. Increases by one month/month when HIV+ |
| Drug resistance | resist_NNRTI | Level of resistance to NNRTI class drugs due to major resistance mutations. | Categorical. Values of 1, medium_‌resist_‌value, and 0, corresponding to none, medium, or high | Varying. Can increase when on an NNRTI class drug |
|  | ‌resist_NRTI | Level of resistance to NRTI class drugs due to major resistance mutations. | Categorical. Values of 1, medium_‌resist_‌value, and 0, corresponding to none, medium, or high | Varying. Can increase when on an NNRTI class drug |
|  | resist_PI | Level of resistance to PI class drugs due to major resistance mutations. | Categorical. Values of 1, medium_‌resist_‌value, and 0, corresponding to none, medium, or high | Varying. Can increase when on an NNRTI class drug |
|  | resist_TAM | Level of resistance to tenofovir and zidovudine due to TAMs | Categorical. Values of 1, medium_‌resist_‌value, and 0, corresponding to none, medium, or high | Varying. Can increase when on zidovudine |
| HIV care | ever_‌tested | Ever tested for HIV? | Categorical. Yes or no | Varying. Can change from no to yes |
|  | time_‌since_‌HIV_‌test | Tracks number of months since last HIV test | Whole number | Varying. Increases by one month/month. Resets to zero upon HIV testing |
|  | time_‌since_‌CD4_‌test | Tracks number of months since last CD4 test | Whole number | Varying. Increases by one month/month. Resets to zero upon CD4 testing |
|  | care_‌status | In HIV care? | Categorical. ‘in care’ or ‘not in care’ | Varying. Can change between yes and no in both directions |
|  | ART_‌status | On ART? | Categorical.  -Never  -1^st^ line ≤1 year  -1^st^ line >1 year  -2^nd^ line ≤1 year  -2^nd^ line >1 year  -dropped out of 1^st^ line  -dropped out of 2^nd^ line | Varying. Explained in section X |
|  | time_‌on_‌ART | Tracks number of continuous months on ART | Whole number | Varying. Increases by one month/month when on ART. Set to zero when not on ART |
|  | time_‌since_‌first_‌starting_‌ART | Tracks number of months since first started ART | Whole number | Varying. Increases by one month/month when on ART or dropped out. |
|  | ever_‌dropped_‌out | Ever dropped out of ART | Categorical. Yes or no | Varying. Can change from no to yes |

Table 2. Partnership state variables

| Name | Description | Type of parameter | Fixed or varying? |
| --- | --- | --- | --- |
| male_‌ID_‌number | The ID number of the man in the partnership | Whole number | Fixed |
| female_‌ID_‌number | The ID number of the woman in the partnership | Whole number | Fixed |
| time_‌left | The maximum remaining lifespan of the partnership in months | Whole number | Varying. Decreases by one month/month |

## Individual attributes/variables calculated from state variables

In addition to the state variables, a number of other individual-level variables are used in the model. Their values can always be calculated from the values of state variables and model-wide parameters, however they are used to make the model more intuitive to understand and faster to run. One example is resist_ART, which indicates an individual’s overall level of resistance to the drug regimen they are taking. Including resist_ART as an individual-level variable means that it can be used throughout a submodel, and only needs to be recalculated when the values of any of the state variables or model-wide parameters used in calculating it change. For very simple variables, the formula is given in the rightmost column of Table 3. For more complicated variables, the table gives the name of the submodel or section that contains the formula. To simplify the model description, we do not always indicate in the submodel pseudo-code where the values of individual-level variables that are not state variables are updated. In the actual model, they are updated whenever the values of the underlying state variables or model-wide parameters are changed, and/or before each use in the model.

Table 3. Individual attributes

| Class | Name | Description | Type of parameter | Fixed or varying? | Formula |
| --- | --- | --- | --- | --- | --- |
| Behaviour | concurrency_‌‌parameter | The state variable concurrency_‌group records whether an individual is in the high or low concurrency group. This parameter records the tendency towards concurrency associated with the group and gender. | Categorical. Equal to high_‌concurrency_‌parameterM, low_‌concurrency_‌parameterM, high_‌concurrency_‌parameterF, or low_‌concurrency_‌parameterF | Varying. Generally fixed, but can change during the two simulated step changes in risk behaviour. | if sex = ‘male’ and concurrency_‌group = ‘high’ [high_‌concurrency_‌parameterM]  if sex = ‘male’ and concurrency_‌group = ‘low’ [low_‌concurrency_‌parameterM]  if sex = ‘female’ and concurrency_‌group = ‘high’ [high_‌concurrency_‌parameterF]  if sex = ‘female’ and concurrency_‌group = ‘low’ [low_‌concurrency_‌parameterF] |
|  | adherence_‌‌adjustment | The state variable *adherence_‌group* records whether an individual is in the high or low adherence group. This parameter records the tendency towards reduced care seeking behaviour and adherence to care associated with the group. | Categorical. Equal to 1, or *start_‌rate_‌low_‌adherence* | Varying. Can change from 1 to *start_‌rate_‌low_‌adherence* when first starting ART. Otherwise, fixed. | if adherence_‌group = ‘high’ [1]  if adherence_‌group = ‘low’ [start_‌rate_‌low_‌adherence] |
| HIV related | HIV_‌‌transmission_‌‌prob | The individual-level per sex act HIV transmission probability | Rational number between 0 and 1 | Varying | See section ‘Transmit HIV’ |
|  | gender_‌‌transmission | The HIV transmission probability modifier associated with their gender | Rational number > 0 | Fixed | See Table 4 |
| Drug resistance | resist_‌‌increased_‌‌mortality | Gives any increase in HIV mortality rate for an individual on ART with resistance to the drug regimen that they are taking | Rational number ≥1 | Varying | See section ‘Update_‌resist_‌increased_‌mortality’ |
|  | resist_‌‌increased_‌‌transmit | Gives any increase in the HIV transmission probability for an individual on ART with resistance to the drug regimen that they are taking | Rational number ≥1 | Varying | See section ‘Update_‌resist_‌increased_‌transmission’ |
|  | resist_‌‌TDF | Gives the level of resistance to tenofovir (TDF) | Rational number between 0 and 1 inclusive | Varying | See section ‘Transmit HIV’ |
|  | resist_‌‌ART | Gives the overall level of resistance a person has to the drug regimen that they are on | Rational number between 0 and 1 inclusive | Varying | See section ‘Update_‌resist_‌ART’ |
|  | individual_‌‌resist_‌‌transmission_‌‌prob | Gives the probability that the person will transmit any drug resistance mutations to anyone who they infect with HIV | Rational number between 0 and 1 inclusive | Varying | See section ‘Transmit HIV’ |
|  | any_‌‌resistance | Records whether an individual has any drug resistance to any of the simulated HIV drugs | Categorical. Yes or no | Varying | See section ‘Update_‌resist_‌ART’ |
|  | resist_‌‌gain_‌‌current_‌‌resist | The rate of developing further resistance increases as the number of active drugs an individual is on declines. This parameter records the size of that increase for the individual. | Rational number ≤1 | Varying | See section ‘Update_‌resist_‌ART’ |
|  | CD4_‌group | Converts the continuous variable *CD4_count*  and the categorical variable *primary_infection* into a categorical variable, which is used for determining mortality rates and transmission probabilities | Categorical 1-9 | Varying | See section ‘Calculate_CD4_group’ |

## Input parameters varied during model fitting

The model has a large number of input parameters used in fitting the model. Table 4 gives a brief description of each input and the plausible range used in model fitting. In some cases, the inputs do not correspond directly to any ‘real life’ values or processes, or no data are available on plausible range. In these cases, very large plausible ranges were used in model fitting, and the fitted range was constrained by model outputs. In other cases, plausible input ranges were based on the 95% confidence intervals from empirical studies. Sources for these data are given in the final column of the table. A fuller explanation of the studies and data on which plausible ranges for inputs were based is given in the supporting methods.

Table 4. Model input parameters.

|  | **Name** | **Description** | **Plausible range** | **Additional constraints** | **Source/justification** |
| --- | --- | --- | --- | --- | --- |
| demographic | birth_‌rate | Number of new people created/woman alive 15 years ago/month | 0.0079 to 0.024 |  | World Population Prospects 2012[1]. Constrained by model outputs. |
| sexual behaviour | partnership_‌duration_‌mean | Mean partnership duration (months) | 3.1 to 12 |  | MRC rural cohort |
|  | risk_‌mix_‌prob | Assortative mixing index | 0.2 to 1 |  | Consistent with high levels of assortative mixing to no assortative mixing. |
|  | prob_‌choosing_‌low_‌riskF | At lower values, both low and high risk men are relatively more likely to form partnerships with high risk women | 0 to 1 |  | Constrained by model outputs |
|  | prop_‌low_‌riskM_‌high_‌concurrency | Proportion of low risk men who are in the high concurrency group | 0 to 1 |  | Constrained by model outputs |
|  | prop_‌high_‌riskF_‌high_‌concurrency | Proportion of high risk women who are in the high concurrency group | 0 to 1 |  | Constrained by model outputs |
|  | high_‌risk_‌contact_‌rateM_‌1 | Contact rate for high risk men in the 1st period (per month) | 0 to 1 |  | Constrained by model outputs |
|  | low_‌risk_‌contact_‌rateM_‌1 | Contact rate for low risk men in the 1st period (per month) | 0 to 1 | < high_‌risk_‌contact_‌rateM_‌1 | Constrained by model outputs |
|  | high_‌risk_‌contact_‌rateM_‌2 | Contact rate for high risk men in the 2nd period (per month) | 0 to 1 |  | Constrained by model outputs |
|  | low_‌risk_‌contact_‌rateM_‌2 | Contact rate for low risk men in the 2nd period (per month) | 0 to 1 | < high_‌risk_‌contact_‌rateM_‌2 | Constrained by model outputs |
|  | high_‌risk_‌contact_‌rateM_‌3 | Contact rate for high risk men in the 3rd period (per month) | 0 to 1 |  | Constrained by model outputs |
|  | low_‌risk_‌contact_‌rateM_‌3 | Contact rate for low risk men in the 3rd period (per month) | 0 to 1 | < high_‌risk_‌contact_‌rateM_‌3 | Constrained by model outputs |
|  | high_‌concurrency_‌parameterM_‌1 | Male high concurrency group concurrency parameter in the 1st risk period | 0 to 1 |  | Constrained by model outputs |
|  | high_‌concurrency_‌parameterF_‌1 | Female high concurrency group concurrency parameter in the 1st risk period | 0 to 1 |  | Constrained by model outputs |
|  | high_‌concurrency_‌parameterM_‌2 | Male high concurrency group concurrency parameter in the 2nd risk period | 0 to 1 |  | Constrained by model outputs |
|  | high_‌concurrency_‌parameterF_‌2 | Female high concurrency group concurrency parameter in the 2nd risk period | 0 to 1 |  | Constrained by model outputs |
|  | high_‌concurrency_‌parameterM_‌3 | Male high concurrency group concurrency parameter in the 3rd risk period | 0 to 1 |  | Constrained by model outputs |
|  | high_‌concurrency_‌parameterF_‌3 | Female high concurrency group concurrency parameter in the 3rd risk period | 0 to 1 |  | Constrained by model outputs |
| HIV mortality | HIV_‌mortality_‌rate_parameter | Determines additional mortality rate/month for HIV+ people who are not on ART | 0.131 to 0.322 |  | Mermin 2008[2] |
|  | reduced_‌mortality_‌in_‌care | Reduction in HIV mortality rate when in pre-ART care | 0.39 to 0.86 |  | Mermin 2008[2] and Uganda Ministry og Health reports[3, 4] |
|  | ARTy2_‌mortality_‌rate_‌parameter | Additional mortality rate/month for HIV+ people on ART >1 year | 0.016 to 0.02 |  | Mills 2011[5] |
|  | ARTy1_‌mortality_‌reduction | Weight used for calculating mortality rates in the 1^st^ year on ARR | 0.5 to 1 |  | Mills 2011[5] |
| HIV care | HIV_‌test_‌rate_‌M_‌1990 | Baseline male HIV testing rate in 1990 (per month) | 0 to 1 |  | Constrained by model output |
|  | HIV_‌test_‌rate_‌F_‌1990 | Baseline female HIV testing rate in 1990 (per month) | 0 to 1 |  |  |
|  | HIV_‌test_‌rate_‌M_‌2005 | Baseline male HIV testing rate in 2005 (per month) | 0 to 1 | ≥ HIV_‌test_‌rate_‌M_‌1990 |  |
|  | HIV_‌test_‌rate_‌F_‌2005 | Baseline female HIV testing rate in 2005 (per month) | 0 to 1 | ≥ HIV_‌test_‌rate_‌F_‌1990 |  |
|  | HIV_‌test_‌rate_‌F_‌2007 | Baseline female HIV testing rate in 2007 (per month) | 0 to 1 | ≥ HIV_‌test_‌rate_‌F_‌2005 |  |
|  | HIV_‌test_‌rate_‌M_‌2012 | Baseline male HIV testing rate in 2012 (per month) | 0 to 1 | ≥ HIV_‌test_‌rate_‌M_‌2007 |  |
|  | HIV_‌test_‌rate_‌F_‌2012 | Baseline female HIV testing rate in 2012 (per month) | 0 to 1 | ≥ HIV_‌test_‌rate_‌F_‌2007 |  |
|  | increased_‌test_‌rate_‌HIV+ | Increased rate of HIV testing in people who are HIV+ | 1 to 10 |  | Constrained by model output |
|  | reduced_‌link_‌to_‌care_‌M | Reduced probability of linking to care after testing positive in men relative to women | 0.5 to 1 |  | Constrained by model output |
|  | link_‌to_‌care_‌F_initial | Probability of linking to care after testing positive before 2008 (female) | 0 to 1 |  | Constrained by model output |
|  | link_‌to_‌care_‌F_‌2008 | Probability of linking to care after testing positive in 2008-2011 (female) | 0 to 1 | ≥ link_‌to_‌care_‌F_initial | Constrained by model output |
|  | link_‌to_‌care_‌F_‌2012 | Probability of linking to care after testing positive after 2012 (female) | 0 to 1 | ≥ link_‌to_‌care_‌F_2008 | Constrained by model output |
|  | prob_‌immediate_‌ART_‌start_initial | Probability of starting ART immediately after tested if eligible, before 2008 | 0 to 1 |  | Constrained by model output |
|  | ‌prob_‌immediate_‌ ART_‌start_‌2008 | Probability of starting ART immediately after tested if eligible, in 2008-2011 | 0 to 1 | ≥ prob_‌immediate_‌ART_‌start_initial | Constrained by model output |
|  | prob_‌immediate_‌ ART_‌start_‌2012 | Probability of starting ART immediately after tested if eligible, after 2012 | 0 to 1 | ≥ prob_‌immediate_‌ART_‌start_2008 | Constrained by model output |
|  | CD4_‌stage3or4_‌slope | Slope of relationship between CD4 count and rate of stage 3/4 clinical events (per month) | -0.0029 to -0.029 |  | Anglaret 2012[6] |
|  | CD4_‌stage3or4_‌multiplier_‌initial | Probability of starting ART (not from care) given stage 3/4 clinical event before 2008 | 0 to 1 |  | Constrained by model output |
|  | increased_‌CD4_‌stage3or4_‌multiplier_‌in_‌care | Increased probability of starting ART given stage 3/4 clinical event when in care | 1 to 10 |  | Constrained by model output |
|  | CD4_‌stage3or4_‌multiplier_‌2008 | Probability of starting ART (not from care) given stage 3/4 clinical event in 2008-2011 | 0 to 1 | ≥ CD4_‌stage3or4_‌multiplier_‌initial | Constrained by model output |
|  | CD4_‌stage3or4_‌multiplier_‌2012 | Probability of starting ART (not from care) given stage 3/4 clinical event after 2012 | 0 to 1 | ≥ CD4_‌stage3or4_‌multiplier_‌2008 | Constrained by model output |
|  | CD4_‌test_‌rate_‌in_‌care_‌initial | Rate of CD4 testing in care when eligible, before 2008 (per month) | 0 to 1 |  | Constrained by model output |
|  | CD4_‌test_‌rate_‌in_‌care_‌2008 | Rate of CD4 testing in care when eligible, in 2008-2011 (per month) | 0 to 1 | ≥ CD4_‌test_‌rate_‌in_‌care_‌initial | Constrained by model output |
|  | CD4_‌test_‌rate_‌in_‌care_‌2012 | Rate of CD4 testing in care when eligible, after 2012 (per month) | 0 to 1 | ≥ CD4_‌test_‌rate_‌in_‌care_‌2008 | Constrained by model output |
|  | pregnancy_‌rate | Rate of pregnancy in women (per month) (used to determine option B+ start rate, not the birth rate) | 0.014 to 0.016 |  | World Population Prospects 2012[1] |
|  | B+_‌coverage | Option B+ coverage (from 2014) | 0 to 1 |  | Constrained by model output |
|  | proportion_‌observing_‌new_‌thresholds | Reduced rates of starting ART between old and new CD4 thresholds, for two years after their introduction | 0 to 1 |  | Constrained by model output |
|  | prob_‌unnecessary_‌switch | Probability of low adherence people on 1st line ART being switched to 2nd line (per month) | 0 to 1 |  | Constrained by model output |
|  | max_‌necessary_‌switch_‌rate | Probability of person with maximum resistance to 1st line ART being switched to 2nd line (per month) | 0 to 1 |  | Constrained by model output |
| adherence and retention | prop_‌high_‌riskM_high_‌adherence | Proportion of men in the high risk group who are assigned to the high adherence group at model entry | 0.5 to 0.9 |  | See supporting methods |
|  | prop_‌low_‌riskM_high_‌adherence | Proportion of men in the low risk group who are assigned to the high adherence group at model entry | 0.8 to 1 |  |  |
|  | dropout_‌rateM | Rate of dropping out of ART for high adherence men (per month) | 0 to 1 |  | Constrained by model output |
|  | dropout_‌rateF | Rate of dropping out of ART for high adherence women (per month) | 0 to 1 |  | Constrained by model output |
|  | increase_‌dropout_‌y1 | Increased rate of dropping out during first year on ART | 1 to 2 |  | Kranzer 2010[7] |
|  | care_‌dropout_‌multiplier | Rate of dropping out of pre-ART care relative to rate of dropping out of ART | 1 to 3 |  | See supporting methods |
|  | restart_‌rateM | Rate of restarting ART for high adherence men (per month) | 0 to 1 |  | Constrained by model output |
|  | restart_‌rateF | Rate of restarting ART for high adherence women (per month) | 0 to 1 |  | Constrained by model output |
|  | adherence_‌adjustment | Relative reduction in care seeking behaviour/adherence/retention for low adherence people compared to high adherence people | 0.25 to 1 |  | See supporting methods |
|  | prob_‌low_‌adherence_‌if_‌no_‌event | Probability of becoming low adherence on starting ART if not starting due to stage 3/4 clinical event | 0 to 0.5 |  | See supporting methods |
|  | increase_‌CD4_‌dropout_‌y1 | Decrease/increase in CD4 count on dropping out of ART after less than one continuous year | -50 to +100 |  | See supporting methods |
|  | increase_‌CD4_‌dropout_‌y2 | Decrease/increase in CD4 count on dropping out of ART after more than one continuous year | 0 to 200 | ≥ increase_‌CD4_‌dropout_‌y1 | See supporting methods |
|  | max_‌ART_‌CD4 | Maximum CD4 count someone can have upon dropping out of ART | 400 to 700 |  | See supporting methods |
|  | medium_‌resist_‌value | Reduction in response to a drug due to medium level drug resistance to that class of drugs | 0.25 to 0.75 |  | See supporting methods |
|  | NRTI_‌weight | Effect of NRTI class drugs relative to NNRTI class drugs | 0.5 to 1 |  | See supporting methods |
|  | PI_‌weight | Effect of PI class drugs relative to NNRTI class drugs | 1 to 2 |  | See supporting methods |
|  | acquire_‌resist_‌rate_‌baseline | Rate of development of resistance to NRTIs for high adherence person with fully active current ART (per month) | 0.0013 to 0.01 |  | Hamers 2012[8], Stadeli 2013[9], Gupta 2008[10] |
|  | reduced_‌acquire_‌resist_‌rate_‌PI | Relative reduction in rate of developing resistance to PI class drugs, relative to NRTI and NNRTI class drugs | 0.05 to 0.5 |  | von Wyl 2007[11] |
|  | reduced_‌acquire_‌resist_‌rate_‌if_‌PI_‌present | Relative reduction in rate of developing resistance if taking a drug regimen containing a PI class drug | 0.25 to 0.75 |  | von Wyl 2007[11] |
|  | acquire_‌resist_‌increase_‌low_‌adherence | Relative increase in rate of resistance development for people in the low adherence group | 1.8 to 11 |  | Hamers 2012[8] |
|  | current_‌resist_‌resist_‌gain_‌link | Increase in rate of resistance development with 2 active drugs vs 3 active drugs (1st line only) | 1.6 to 3.4 |  | Hamers 2012[8], von Wyl 2007[11] |
|  | increased_‌NRTI_‌resist_‌decline | Reduced probability of transmitting NRTI mutations (compared to other mutations) when not on ART | 0.5 to 1 |  | Jain 2011[12] |
|  | prob_‌full_‌NNRTI_‌resist | Probability of immediately developing high NNRTI resistance, bypassing medium resistance | 0.5 to 1 |  | See supporting methods |
| HIV transmission | baseline_‌transmission | Mean (by gender) HIV transmission probability for people with CD4 counts of 200-350 cells/µL | 0 to 1 |  | Constrained by model output |
|  | m2f_‌relative_‌to_‌f2m | Ratio of male->female to female->male transmission probabilities | 1.1 to 4.8 |  | Nicolosi 1994[13] |
|  | onART_‌transmission | Relative reduction in HIV transmission probabilities while on ART (relative to CD4 200-350 cells/µL) | 0.04 to 0.21 |  | Baggaley 2013[14] |
|  | HIV_‌transmission_‌primary | Ratio of transmission probabilities with primary stage infection to transmission probabilities with CD4 counts of 200-350 | 1 to 60 | ln(primary_‌time_‌mean) < -1.47 * ln(HIV_‌transmission_‌primary) + 6 | Bellan 2015[15] |
|  | CD4<200_‌transmission | Ratio of transmission with CD4<200 cells/µL to transmission with a CD4 count of 200-350 cells/µL | 1.5 to 6.7 |  | Donnell 2010[16] |
|  | CD4>350_‌transmission | Ratio of transmission with CD4>350 cells/µL to transmission with a CD4 count of 200-350 cells/µL | 0.42 to 0.95 |  |  |
|  | resist_‌transmission_‌1 | Probability of transmitting a resistance mutation for a person not on ART with CD4_‌group = 1 | 0.9 to 1 |  | Kaleebu 2001[17] and Castro 2013[18] |
|  | resist_‌transmission_‌2 | Probability of transmitting a resistance mutation for a person not on ART with CD4_‌group = 2 | 0.84 to 0.93 | < resist_‌transmission_‌1 |  |
|  | resist_‌transmission_‌3 | Probability of transmitting a resistance mutation for a person not on ART with CD4_‌group = 3 | 0.41 to 0.77 | < resist_‌transmission_‌2 |  |
|  | resist_‌transmission_‌4 | Probability of transmitting a resistance mutation for a person not on ART with CD4_‌group = 4 | 0.14 to 0.63 | < resist_‌transmission_‌3 |  |
|  | resist_‌transmission_‌5 | Probability of transmitting a resistance mutation for a person not on ART with CD4_‌group = 5 | 0.067 to 0.54 | < resist_‌transmission_‌4 |  |
|  | resist_‌transmission_‌6 | Probability of transmitting a resistance mutation for a person not on ART with CD4_‌group = 6 | 0.039 to 0.48 | < resist_‌transmission_‌5 |  |
|  | resist_‌transmission_‌7 | Probability of transmitting a resistance mutation for a person not on ART with CD4_‌group = 7 | 0.02 to 0.42 | < resist_‌transmission_‌6 |  |
|  | resist_‌transmission_‌8 | Probability of transmitting a resistance mutation for a person not on ART with CD4_‌group = 8 | 0.009 to 0.36 | < resist_‌transmission_‌7 |  |
|  | resist_‌transmission_‌9 | Probability of transmitting a resistance mutation for a person not on ART with CD4_‌group = 9 | 0.00049 to 0.3 | < resist_‌transmission_‌8 |  |
|  | resist_‌transmission_‌ART | Probability of transmitting a resistance mutation for a person on ART with reduced activity due to that mutation | 0.9 to 1 | ≥ resist_‌transmission_‌1 | See supporting methods |
|  | resist_‌transmission_‌other_‌ART | Probability of transmitting a resistance mutation for person on ART that does not have reduced activity due to that mutation | 0 to 0.4 |  | See supporting methods |
|  | resist_‌transmission_‌dropout | Probability of transmitting a resistance mutation for a person who has dropped out of ART | 0.05 to 0.4 |  | Kranzer 1999[7] and Castro 2013[18] |
| HIV natural history | primary_‌time_‌mean | Mean duration of primary infections (months) | 1 to 8 | ln(primary_‌time_‌mean) < -1.47 * ln(HIV_‌transmission_‌primary) + 6 | Bellan 2015[15] |
|  | CD4_‌decline_‌mean | Mean decline in root CD4 per year (√cells/µL, converted to per month in model) | 0.73 to 2.2 |  | Kaleebu 2001[17] |
| Seeding | seed_‌resist_‌2003 | Proportion of HIV+ people seeded with drug resistance in 2003 | 0 to 0.1 |  | See supporting methods |
|  | seed_‌HIV_‌low_‌risk | Proportion of low risk people seeded with HIV in 1970 | 0.001 to 0.05 | < seed_‌HIV_‌high_‌risk |  |
|  | seed_‌HIV_‌high_‌risk | Proportion of high risk people seeded with HIV in 1970 | 0.001 to 0.1 |  |  |

## Other model-wide parameters

Table 5. Model-wide parameters

|  | **Name** | **Description** | **Value** | **Source** |
| --- | --- | --- | --- | --- |
| Demographic | background_‌mortalityM | Non-HIV mortality rate for men/month | 0.00070 | World Population Prospects 2012[1] |
|  | background_‌mortalityF | Non-HIV mortality rate for women/month | 0.00059 | World Population Prospects 2012[1] |
|  | max_age | Maximum age that people can reach in the model, in months from age 15 years | 420 | Gives a maximum age of 49 years |
|  | women_‌15y_‌ago | Gives the number of women in the model 15 years ago | Varying |  |
|  | women_‌15y_‌ago_list | Tracks the number of women in the model at the end of the year for the preceding 15 years | Varying |  |
| Sexual behaviour | prop_‌high_‌riskM | Proportion of men who are in the high risk sexual behaviour group | 0.21 | MRC rural cohort. See supporting methods |
|  | prop_‌high_‌riskF | Proportion of women who are in the high risk sexual behaviour group | 0.15 | MRC rural cohort. See supporting methods |
|  | prop_‌high_‌riskM_‌high_‌concurrency | Proportion of high risk men who are in the high concurrency group | 1 | It is assumed that all high risk men are capable of forming concurrent partnerships, and they are therefore all placed into the high concurrency group. |
|  | prop_‌low_‌riskF_‌high_‌concurrency | Proportion of low risk women who are in the high concurrency group | 0 | It assumed that low risk women do not form concurrent partnerships, and they are therefore all placed into the low concurrency group |
|  | low_‌concurrency_‌parameterM | Male low concurrency group concurrency parameter | 0 | It is assumed that men in the low concurrency group do not have concurrent partners |
|  | low_‌concurrency_‌parameterF | Female low concurrency group concurrency parameter | 0 | It is assumed that women in the low concurrency group do not have concurrent partners |
|  | high_‌risk_‌contact_‌rateM | Contact rate for high risk men | Varying | The parameter values can be different in each of the three risk behaviour periods. Input parameters in Table 4 give plausible ranges for the parameter values in each of the three risk periods. |
|  | low_‌risk_‌contact_‌rateM | Contact rate for high low men | Varying |  |
|  | high_‌concurrency_‌parameterM | Male high concurrency group concurrency parameter | Varying |  |
|  | high_‌concurrency_‌parameterF | Female high concurrency group concurrency parameter | Varying |  |
|  | prob_high_riskM_high_riskW | Probability that a high risk man forming a new partnership will choose a high risk woman | Varying |  |
|  | prob_low_riskM_low_riskW | Probability that a low risk man forming a new partnership will choose a low risk woman | Varying |  |
|  | partnership_‌duration_‌SD | Standard deviation of partnership durations (months) | partnership_‌duration_‌mean/5 |  |
| HIV | initial_‌CD4_‌meanM | Mean CD4 count in men on infection with HIV (cells/µL) | = 11.831 * CD4_‌decline_‌mean + 542.39 | See supporting methods |
|  | initial_‌CD4_‌meanF | Mean CD4 count in women on infection with HIV (cells/µL) | = 12.605 * CD4_‌decline_‌mean + 616.89 | See supporting methods |
|  | initial_‌CD4_‌SD | Standard deviation for CD4 count on infection with HIV (cells/µL) | 71 | See supporting methods |
|  | CD4_‌decline_‌SD | Standard deviation for decline in root CD4 per year (√cells/µL, converted to per month in model) | CD4_‌decline_‌mean/5 | See supporting methods |
|  | primary_‌time_‌SD | Standard deviation for duration of primary infections (months) | primary_‌time_‌mean/5 | See supporting methods |
| HIV transmission | HIV_‌transmission_2 | Ratio of transmission with CD4>500 cells/µL to transmission with a CD4 count of 200-350 cells/µL | CD4>350_‌transmission | See supporting methods |
|  | HIV_‌transmission_3 | Ratio of transmission with CD4 350-500 cells/µL to transmission with a CD4 count of 200-350 cells/µL | CD4>350_‌transmission | See supporting methods |
|  | HIV_‌transmission_4 | CD4 250-350 cells/µL is within the range used to calculate the baseline HIV transmission probability, therefore this is equal to 1. | 1 | See supporting methods |
|  | HIV_‌transmission_5 | CD4 200-250 cells/µL is within the range used to calculate the baseline HIV transmission probability, therefore this is equal to 1. | 1 | See supporting methods |
|  | HIV_‌transmission_6 | Ratio of transmission with CD4 150-200 cells/µL to transmission with a CD4 count of 200-350 cells/µL | CD4<200_‌transmission | See supporting methods |
|  | HIV_‌transmission_7 | Ratio of transmission with CD4 100-150 cells/µL to transmission with a CD4 count of 200-350 cells/µL | CD4<200_‌transmission | See supporting methods |
|  | HIV_‌transmission_8 | ratio of transmission with CD4 50-100 cells/µL to transmission with a CD4 count of 200-350 cells/µL | CD4<200_‌transmission | See supporting methods |
|  | HIV_‌transmission_9 | ratio of transmission with CD4 <50 cells/µL to transmission with a CD4 count of 200-350 cells/µL | CD4<200_‌transmission | See supporting methods |
| HIV care | HIV_‌‌test_‌‌rate_‌‌M_‌2007 | Baseline male HIV testing rate in 2007 (per month) | HIV_‌‌test_‌‌rate_‌‌F_‌2007 * HIV_‌‌test_‌‌rate_‌‌M_‌2012 / HIV_‌‌test_‌‌rate_‌‌F_‌2012 | See supporting methods |
|  | HIV_‌test_‌rate_‌M | Baseline HIV testing rate in men | Varying | The parameter values can be different in different model years. Input parameters in Table 4 gives plausible ranges for the parameter values in each year. |
|  | HIV_‌test_‌rate_‌F | Baseline HIV testing rate in women | Varying |  |
|  | link_‌to_‌care_‌M | Probability of linking to care after testing positive (male) | Varying |  |
|  | link_‌to_‌care_‌F | Probability of linking to care after testing positive (female) | Varying |  |
|  | prob_‌immediate_‌ART_‌start | Probability of immediately starting ART if linked to care | Varying |  |
|  | CD4_‌stage3or4_‌multiplier | Probability of starting ART (not from care) given stage 3/4 clinical event | Varying |  |
|  | CD4_‌in_‌care_‌stage3or4_‌multiplier | Probability of starting ART (from care) given stage 3/4 clinical event | Varying |  |
|  | CD4_test_rate_in_care | Rate of CD4 testing in care when eligible (per month) | Varying |  |
|  | CD4_‌threshold | CD4 threshold for starting ART based on national policy, and observed by some clinics | Varying | Uganda National guidelines[19]. See supporting methods |
|  | effective_CD4_‌threshold | Lower CD4 threshold for starting ART, and minimum level observed by all clinics | Varying | See supporting methods |
|  | max_‌total_‌ART | Maximum number of active drugs that a person can be on, after adjusting for relative effects of different drug classes | Fixed | See section ‘Initialisation’ |
|  | prop_‌high_‌riskF_high_‌adherence | Proportion of women in the high risk group who are assigned to the high adherence group at model entry | prop_‌high_‌riskM_‌high_‌adherence | See supporting methods |
|  | prop_‌low_‌riskF_high_‌adherence | Proportion of women in the low risk group who are assigned to the high adherence group at model entry | prop_‌low_‌riskM_‌high_‌‌adherence | See supporting methods |

## Initialisation pseudo-code

get_inputs #this submodel reads in the values of the model input parameters from a .csv input file. Details of the submodel are not given in this document.

#The majority of parameter values are read directly from the input file. A number are defined in the model itself however, or are calculated from parameters in the input file. Details of these are given below.

set year 1950

set max_age (12 * 35) #the maximum age in the model is 50 years. However, as people enter the model at age 15, this gives a maximum age of (50 – 15) = 35 years.

set m2f_transmission (2 * baseline_transmission / (1 + 1 / m2f_relative_to_f2m))

set f2m_transmission (2 * baseline_transmission / (m2f_relative_to_f2m + 1))

set CD4_decline_mean (CD4_decline_mean / 12) #this value is given as an annual rate in the input file, and so is converted to a monthly rate during model initialisation

set CD4_decline_SD (CD4_decline_SD / 12) #this value is given as an annual rate in the input file, and so is converted to a monthly rate during model initialisation

set max_total_ART min (1 + 2 * NRTI_weight), (PI_weight + 2 * NRTI_weight)

set women_15y_ago 600

set women_15y_ago_list [600, 600, 600, 600, 600, 600, 600, 600, 600, 600, 600, 600, 600, 600]

set HIV_test_rate_M HIV_‌test_‌rate_‌M_‌1990

set HIV_test_rate_F HIV_‌test_‌rate_‌F_‌1990

set link_to_care_F link_‌to_‌care_‌F_initial

set link_to_care_M (link_‌to_‌care_‌F_initial * reduced_link_to_careM)

set prob_‌immediate_‌ART_‌start prob_‌immediate_‌ART_‌start_initial

set CD4_‌stage3or4_‌multiplier CD4_‌stage3or4_‌multiplier_initial

set CD4_‌in_‌care_‌stage3or4_‌multiplier (CD4_‌stage3or4_‌multiplier_initial *

increased_CD4_stage3or4_multiplier_in_care)

set CD4_test_rate_in_care CD4_test_rate_in_care_initial

#the lines below create the initial people in the model. See section ‘birth’ for more details

create 612 **men**

create 612 **women**

ask **people** [

set age random-uniform [35 * 12)

set CD4_count random-normal(initial_CD4_meanM/F, initial_CD4_SD)

set CD4_decline_rate random-normal(CD4_decline_mean, CD4_decline_SD)

set primary_time random-normal(primary_time_mean, primary_time_SD)

ifelse random-uniform [0,1) < prop_high_riskM/F

then [

set risk_group ‘high’

ifelse random-uniform [0,1) < prop_high_riskM/F_high_concurrency

then [set concurrency_group ‘high’]

otherwise [set concurrency_group ‘low’]

ifelse random-uniform [0,1) < prop_‌high_‌riskM/F_high_‌adherence

then [set adherence_group ‘high’]

otherwise [set adherence_group ‘low’]

]

otherwise [

set risk_group ‘low’

ifelse random-uniform [0,1) < prop_low_riskM/F_high_concurrency

then [set concurrency_group ‘high’]

otherwise [set concurrency_group ‘low’]

ifelse random-uniform [0,1) < prop_‌low_‌riskM/F_high_‌adherence

then [set adherence_group ‘high’]

otherwise [set adherence_group ‘low’]

]

]

## Process overview and scheduling

Table 6 lists the schedule of model processes. The processes are run through in order each time step (i.e. each month)

Table 6. Schedule of model processes

| Name | Description | Timing |
| --- | --- | --- |
| Second year ART | Change people’s ART_status from ≤1 year on ART to >1 year | Every month |
| Track times | Update variables tracking time in state (e.g. time_since_infection) | Every month |
| Mortality | Kill people and remove them from the model | Every month |
| Birth | Create new people | Every month |
| Update partnerships | Start and end partnerships | If year ≥1965 |
| Update CD4 count | Reduce CD4 count for HIV+ people who are not on ART | If year ≥1970 |
| Transmit HIV | Transmit HIV within partnerships | If year ≥1970 |
| Drop out care | Move people from in care to not in care | If year ≥2003 |
| HIV test | Test people for HIV | If year ≥1990 |
| Track ART | Track decline in CD4 count | If year ≥2003 |
| Restart ART | Restart people on ART | If year ≥2003 |
| Drop out ART | Remove people from ART | If year ≥2003 |
| Start ART | Start people on ART | If year ≥2003 |
| Switch to 2^nd^ line | Switch people from 1^st^ line to 2^nd^ line ART | If year ≥2003 |
| Develop resistance | Increase the drug resistance of people on ART | If year ≥2003 |
| Annual output | Record output variables for that year | if month mod 12 = 0 |
| Change year | Track the model year | if month mod 12 = 0 |
| Scale up ART | Update parameter values determining rates of HIV testing and starting ART | if month mod 12 = 0 |
| Change risk behaviour | Change parameter values determining risk behaviour | if month mod 12 = 0 |
| Interventions | Implement interventions | if (year = 2016 or year = 2017 or year = 2018) and month mod 12 = 0 |
| Seed HIV | Make a proportion of people HIV+ | if year = 1970 and month mod 12 = 0 |
| Seed resistance | Make a proportion of HIV+ people have medium resistance to NNRTI class drugs | if year = 2003 and month mod 12 = 0 |
| Count women | Track the number of women in the model | if month mod 12 = 0 |
| Reset lists | A number of lists are used to track the number of events which occur each year (e.g. new HIV infections in women). These lists are emptied at the end of each year. Details of this are not given. | if month mod 12 = 0 |

For processes which are conducted separately for each person or partnership in the model, the order in which the entities conduct the process is random.

## Submodels

In this section we give a brief description of what each submodel does, followed by pseudo-code for the submodel.

### Second year ART

#### Description

In the model, ART drop out rates and mortality rates fall after a person has spent one year or more continuously on ART. This submodel changes people’s ART status from less than one year to greater than one year after they have spent a year continuously on ART.

#### Process

ask **people** with (time_‌on_‌ART = 12 and ART_‌status = ‘1^st^ line <12 months’) [

set ART_‌status ‘1^st^ line >12 months’

]

ask **people** with (time_‌on_‌ART = 12 and ART_‌status = ‘2^nd^ line <12 months’) [

set ART_‌status ‘2^nd^ line >12 months’

]

### Track times

#### Description

People in the model have memories of number of months spent in certain states (e.g. HIV+). This submodel increases these by one month each month

#### Process

ask **people** [

set age (age + 1)

]

ask **people** with (HIV_‌status = ‘positive’) [

set time_‌since_‌infection (time_‌since_‌infection + 1)

]

ask **people** with (ART_‌status = ‘on ART’) [

set time_‌on_‌ART (time_‌on_‌ART + 1)

]

ask **people** with (ART_‌status = ‘on ART’ or ‘dropped out’) [

set time_‌since_‌first_‌starting_‌ART (time_‌since_‌first_‌starting_‌ART + 1)

]

### Mortality

#### Description

There are three categories of mortality in the model: background mortality, old age mortality, and HIV mortality. Background mortality represents all mortality due to causes other than HIV between the ages of 15 and 49 years. Background mortality rates are different for men and women, but are the same for all people in the model of the same gender. The model only represents individuals aged 15-49 years. Old age mortality instantly removes people form the model when they reach the age of 50 years. HIV mortality is broken down into pre-ART not in care mortality, pre-ART in care mortality, mortality on ART ≤1 year, mortality on ART >1 year and drop out mortality. Partnerships end automatically when one of the partners die. At the end of the submodel, people remaining alive are asked to recalculate their availability to form new partnerships.

#### Process

1. **Background mortality**

ask **men** [

if random-uniform [0,1) < background_mortalityM

then [die]

]

ask **women** [

if random-uniform [0,1) < background_mortalityM

then [die]

]

1. **Old age mortality**

ask **people** with (age > max_age) [

die

]

1. **HIV mortality**

ask **people** with (ART_‌status = ‘never’ and care_status = ‘not in care’) [

if random-uniform [0,1) < HIV_mortality_rate_parameter * exp(-0.0141 * CD4_count)

then [die]

]

ask **people** with ART_‌status = ‘never’ and care_status = ‘in care’ [

if random-uniform [0,1) < HIV_mortality_rate_parameter * exp(-0.0141 * CD4_count) * reduced_mortality_jn_care

then [die]

]

ask **people** with (ART_‌status = ‘ART ≤1 year’) [

Update_‌resist_‌increased_‌mortality

if random-uniform [0,1) < resist_‌increased_‌mortality * min (ARTy1_mortality_reduction * (-0.00232 * ln(CD4_count) + ARTy2_mortality_rate_parameter) + (1 - ARTy1_mortality_reduction) * (HIV_mortality_rate_parameter * exp(-0.0141 * CD4_count) * reduce_mortality_in_care)), ((HIV_mortality_rate_parameter * exp(-0.0141 * CD4_count)) * reduce_mortality_in_care)

then [die]

]

ask **people** with ART_‌status = ‘ART >1 year’ [

Update_‌resist_‌increased_‌mortality

if random-uniform [0,1) < resist_‌increased_‌mortality * min (-0.00232 * ln(CD4_count) + ARTy2_mortality_rate_parameter), ((HIV_mortality_rate_parameter * exp(-0.0141 * CD4_count)) * reduce_mortality_in_care)

then [die]

]

ask **people** with ART_‌status = ‘dropped out’ [

if random-uniform [0,1) < HIV_mortality_rate_parameter * exp(-0.0141 * CD4_count)

then [die]

]

1. ask **people** [

**Recalculate_‌availability**

]

### Birth

#### Description

As people are introduced into the model at age 15, the number of people introduced each month is related to the number of women alive 15 years ago. Upon creation, new people are assigned a model age of 0 months (equivalent to a real life age of 15 years), a CD4 count, a CD4 decline rate, and a length of primary stage infection in months. The latter three characteristics are assigned at model entry, but have no effect unless and until the person is infected with HIV. In addition to this, people are assigned at model entry to a sexual behaviour risk group (high or low), a partnership concurrency group (high or low), and an adherence group (high or low). The probability of being assigned to each group is different for men and women, and the probability of being assigned to the high concurrency group and/or the low adherence group is higher for men and women in the high risk sexual behaviour group.

#### Process

create N_m_ **men**, where N_m_ = random-binomial(women_‌15y_‌ago, (birth_‌rate / 2))

create N_w_ **women**, where N_w_ = random-binomial(women_‌15y_‌ago, (birth_‌rate / 2))

ask **new (wo)men** [

set age = 0

set CD4_‌count random-normal(initial_‌CD4_‌mean, initial_‌CD4_‌SD)

set CD4_‌decline_‌rate random-normal(CD4_‌decline_‌mean, CD4_‌decline_‌SD)

set primary_‌time random-normal(primary_‌time_‌mean, primary_‌time_‌SD)

ifelse random-uniform [0,1) < prop_‌high_‌riskM/F

then [

set risk_‌group ‘high’

ifelse random-uniform [0,1) < prop_‌high_‌riskM/F_‌high_‌concurrency

then [set concurrency_‌group ‘high’]

otherwise [set concurrency_‌group ‘low’]

ifelse random-uniform [0,1) < prop_‌high_‌riskM/F_high_‌adherenceF

then [set adherence_‌group ‘high’]

otherwise [set adherence_‌group ‘low’]

]

otherwise [

set risk_‌group ‘low’

ifelse random-uniform [0,1) < prop_‌low_‌riskM/F_‌high_‌concurrency

then [set concurrency_‌group ‘high’]

otherwise [set concurrency_‌group ‘low’]

ifelse random-uniform [0,1) < prop_‌low_‌riskM/F_high_‌adherence

then [set adherence_‌group ‘high’]

otherwise [set adherence_‌group ‘low’]

]

]

### Update partnerships

#### Description

There are two steps to this submodel, starting partnerships and ending partnerships. Partnerships also end automatically when either of the partners die. After each partnership starts or ends, both partners recalculate whether they are available for another partnership. People also recalculate whether they are available for another partnership whenever an existing partner dies.

**Start partnerships**

Each month (provided that the model contains sufficient available women), all high and low risk men who are available to start a new partnership can start a new partnership with probability *high_‌risk_‌contact_‌rateM* and *low_‌risk_‌contact_‌rateM* respectively. The probabilities that high and low risk men will choose to form new partnerships with someone in their own risk group (*prob_high_riskM_high_riskW* and *prob_low_riskM_low_riskW* respectively) are calculated the start of the submodel. The probabilities depend on the number of available high and low risk women, a mixing parameter (*risk_‌mix_‌prob*), and a parameter which can alter the overall probability that a low risk woman is chosen (*prob_‌choosing_‌low_‌riskF*). *Risk_‌mix_‌prob* determines the overall level of mixing between risk groups. When it is set at 0, men cannot chose partners outside their own risk group. When it is set at 1, men have no preference for or against women in their own risk group. At intermediate values, men have some preference for women in their own risk group. *Prob_‌choosing_‌low_‌riskF* alters the probability that both high and low risk men choose low risk women. When set to 0, men cannot start partnerships with low risk women. When set to 1, men have no preference for low or high risk women, beyond that determined by their risk group and *risk_‌mix_‌prob*.

When a new partnership is formed, its maximum duration in months is selected from a normal distribution with mean *partnership_‌duration_‌mean* and standard deviation *partnership_‌duration_‌SD*.

**End partnerships**

Each month, one month is removed from each partnerships’ remaining duration. Once the remaining duration falls below half a month, the partnership ends. Partnerships also end automatically when either of the partners die.

#### Process

1. #Starting partnerships

if count **women** (with available = ‘yes’) > 0

then [

set prob_low_riskM_low_riskW ((1 - risk_‌mix_‌prob / 2) * prob_‌choosing_‌low_‌riskF * count **women** (with available = ‘yes’ and risk = ‘low’))) / ((1 - risk_‌mix_‌prob / 2) * prob_‌choosing_‌low_‌riskF * count **women** with available = ‘yes’ and risk = ‘low’ + risk_‌mix_‌prob / 2 * count **women** with (available = ‘yes’ and risk = ‘high’))

set prob_high_riskM_high_riskW ((1 - risk_‌mix_‌prob / 2) * count **women** with (available = ‘yes’ and risk = ‘high’)) / ((1 - risk_‌mix_‌prob / 2) * count **women** with (available = ‘yes’ and risk = ‘high’) + risk_‌mix_‌prob / 2 * prob_‌choosing_‌low_‌riskF * count **women** with (available = ‘yes’ and risk = ‘low’))

]

ask **men** with (available = ‘yes’ and risk = ‘low’) [

if random-uniform [0,1) < low_‌risk_‌contact_‌rateM

then [

ifelse random-uniform [0,1) < prob_low_riskM_low_riskW

then [if count **women** (with available = ‘yes’ and risk = ‘low’) > 0

then [

create **partnership** with one-of **women** with (available = ‘yes’ and risk = ‘low’)

ask **new partnership** [*set time_‌left random-normal(partnership_‌duration_‌mean, partnership_‌duration_‌SD)*]

]

]

otherwise [if count **women** with (available = ‘yes’ and risk = ‘high’) > 0

then [

create p**artnership** with one-of **women** with (available = ‘yes’ and risk = ‘high’)

ask **new partnership** [*set time_‌left random-normal(partnership_‌duration_‌mean, partnership_‌duration_‌SD)*]

]

]

]

ask **new partner** [*recalculate_‌availability*]

[recalculate_‌availability]

]

ask **men** with (available = ‘yes’ and risk = ‘high’) [

if random-uniform [0,1) < high_‌risk_‌contact_‌rateM

then [

ifelse random-uniform [0,1) < prob_high_riskM_high_riskW

then [if count **women** (with available = ‘yes’ and risk = ‘high’) > 0

then [

create **partnership** with one-of **women** with (available = ‘yes’ and risk = ‘high’)

ask **new partnership** [*set time_‌left random-normal(partnership_‌duration_‌mean, partnership_‌duration_‌SD)*]

]

]

otherwise [if count **women** with (available = ‘yes’ and risk = ‘low’) > 0

then [

create p**artnership** with one-of **women** with (available = ‘yes’ and risk = ‘low’)

ask **new partnership** [*set time_‌left random-normal(partnership_‌duration_‌mean, partnership_‌duration_‌SD)*]

]

]

]

ask **new partner** [*recalculate_‌availability*]

[recalculate_‌availability]

]

1. #Ending partnerships

ask **partnerships** if time_‌left < 0.5 [die]

ask **partnerships** [set time_‌left (time_‌left – 1)]

ask **people** [recalculate_‌availability]

### Update CD4 count

#### Description

This submodel tracks the decline in CD4 count over time for HIV positive people who are not on ART. It also tracks time spent with a primary infection, and changes people’s primary_‌infection parameter from ‘yes’ to ‘no’ after their time with a primary infection has expired.

#### Process

ask **people** with (HIV_‌status = ‘positive’ and ART_‌status = ‘not on ART’) [

ifelse CD4_‌count > 0

then [set CD4_‌count (√CD4_‌count - CD4_‌decline_‌rate)^2^]

otherwise [set CD4_‌count 0]

]

ask **people** with (primary_‌infection = ‘yes’) [

if primary_‌time < 0

then [set primary_‌infection = ‘no’]

set primary_‌time (primary_‌time – 1)

]

### Transmit HIV

#### Description

There are three steps to this submodel:

1. Calculate the per sex act HIV transmission probability for each individual (HIV_‌transmission_‌prob). This is calculated as follows:

- Firstly, there is baseline transmission probability that applies to everyone in the model (baseline_‌transmission).
- Secondly, this is adjusted for the gender of the potential transmitter, as male to female transmission probabilities in the model can be higher than female to male transmission probabilities.
- Thirdly, it is adjusted according to certain characteristics of the potential transmitter:
  - For HIV+ people not on ART (ART naïve and drop outs), transmission probabilities are highest in people with primary infections. For post-primary infections, transmission probabilities increase with decreasing CD4 count.
  - For HIV+ people on ART, transmission probabilities increase as the number of active drugs falls (i.e. with increasing resistance to the regimen that they are on). With the number of active drugs equal to the maximum, the transmission probability is equal to *on_‌ART_‌HIV_‌transmission*. With the number of active drugs equal to 0 (i.e. total resistance), the transmission probability is the same as the transmission probability for a person not on ART with the same CD4 count.

1. For each HIV+ individual who is not on ART, calculate the probability that resistance mutations will be transmitted (assuming that HIV transmission occurs) (individual_‌resistance_‌transmission_‌prob). For ART naïve individuals, this is highest during primary infection, and decreases with decreasing CD4 count. For ART drop outs, there is a single model-wide parameter determining the resistance transmission probability.
2. Transmit HIV.

It is assumed in the model that sex occurs 10 times per month in each partnership. Each time sex occurs between an HIV+ and HIV- couple, transmission can occur. The calculation of the transmission probability is described above.

When HIV is transmitted by a person with one or more drug resistance mutations, then there is a chance that each mutation can be transmitted. This is independent for each mutation. ‘High’ level resistance of any type (eg high NNRTI resistance or high TAM resistance) is assumed to consist of two ‘medium’ level resistance mutations that can be transmitted separately. This means that, for example, if the probability of resistance transmission by a person with high PI resistance is *p*, there is a *p^2^* chance that a person they infect will be infected with a strain with high PI resistance, a *p* - *p^2^* chance that they will be infected with a strain with medium PI resistance, and a *1 – p* chance that they will be infected with a strain with no PI resistance. The probability of transmission is the same for all resistance types, with the exception of NRTI resistance, which can have a lower probability of transmission by people not on ART in the model. For people on ART, the probability of a resistance mutations being transmitted is different depending on whether or not that resistance mutation reduces the activity of the regimen that they are on. For instance, the 1^st^ line regimen contains a NNRTI class drug, but the 2^nd^ line regimen does not. The probability of NNRTI resistance for someone on 1^st^ line ART will therefore be equal to the resist_transmission_onART parameter, and the probability for someone on 2^nd^ line ART will be equal to the resist_transmission_other_ART parameter.

#### Process

1. #Calculate HIV transmission probabilities

ask **men** [set gender_‌transmission m2f_‌transmission]

ask **women** [set gender_‌transmission f2m_‌transmission]

ask **people** with (ART_‌status = ‘on ART’) [

update_‌resist_‌increased_‌transmission

for **‘i’**  in (1, 2, 3, 4, 5, 6, 7, 8, 9) {

set resist_‌increased_‌transmit (exp((1 - resist_‌ART) * ln(HIV_‌transmission_‌***’i'***/ onART_‌HIV_‌transmission)

}

set HIV_‌transmission_‌prob (baseline_‌transmission * gender_‌transmission * onART_‌HIV_‌transmission * resist_‌increased_‌transmit)

]

ask **people** with (HIV_‌status = ‘positive’ and ART_‌status = ‘not on ART’) [

for **‘i’**  in (1, 2, 3, 4, 5, 6, 7, 8, 9) {

if CD4_‌group = ***‘i’***

then [

set HIV_‌transmission_‌prob (baseline_‌transmission * gender_‌transmission * HIV_‌transmission_‌***’i'***)

]

}

]

1. #Calculate individual resistance transmission probabilities

ask **people** with (HIV_‌status = ‘positive’ and ART_‌status = ‘never’) [

for ***‘i’*** in (1, 2, 3, 4, 5, 6, 7, 8, 9) {

if CD4_‌group = ***‘i’*** [set individual_‌resist_‌transmission_‌prob resist_‌transmission_‌***’i'***]

}

]

ask **people** with ART_‌status = ‘dropped out’ [

set individual_‌resist_‌transmission_‌prob resist_transmission_dropout

]

1. #Transmit HIV. This is done separately for people not on ART, people on 1^st^ line ART, and people on 2^nd^ line ART. When someone is not on ART the probability of resistance transmission is determined by individual attributes, as defined above. When someone is on ART, the probability of resistance transmission is determined by whether or not the specific class of mutation reduces the activity of the drug regimen that they are on.

#Not on ART

ask **people** with (HIV_‌status = ‘positive’ and ART_‌status = ‘not on ART’) [

for each **sexual partner** with (*HIV_‌status = ‘negative’*) **{**

if random-binomial(10, HIV_‌transmission_‌prob) > 0

then [

ask **sexual partner** [*set* *HIV_‌status ‘positive’*]

ask **sexual partner** [*set* *primary_‌infection ‘yes’*]

#Transmit drug resistance. Only occurs if HIV transmission occurs

if resist_NNRTI ≠ ‘none’ and random-uniform [0,1) < individual_‌resist_‌transmission_‌prob

then [

ask **sexual partner** [*set resist_NNRTI ‘medium’*]

#as the step below is only executed if the condition above is met, it gives an overall probability of high NNRTI resistance being transmitted of individual_‌resist_‌transmission_‌prob^2^

if resist_NNRTI = ‘high’ and random-uniform [0,1) <

individual_‌resist_‌transmission_‌prob

then [ask **sexual partner** [*set* *resist_NNRTI ‘high’*]]

]

if resist_NRTI ≠ ‘none’ and random-uniform [0,1) < individual_‌resist_‌transmission_‌prob * reduced_‌NRTI_‌resist_‌transmit

then [

ask **sexual partner** [*set resist_NRTI ‘medium’*]

if resist_NRTI = ‘high’ and random-uniform [0,1) <

individual_‌resist_‌transmission_‌prob * reduced_‌NRTI_‌resist_‌trans

then [ask **sexual partner** [*set resist_NRTI ‘high’*]]

]

if resist_PI ≠ ‘none’ and random-uniform [0,1) < individual_‌resist_‌transmission_‌prob

then [

ask **sexual partner** [*set resist_PI ‘medium’*]

if resist_PI = ‘high’ and random-uniform [0,1) < individual_‌resist_‌transmission_‌prob

then [ask **sexual partner** [*set* *resist_PI ‘high’*]]

]

if resist_TAM≠ ‘none’ and random-uniform [0,1) < individual_‌resist_‌transmission_‌prob

then [

ask **sexual partner** [*set resist_TAM ‘medium’*]

if resist_TAM= ‘high’ and random-uniform [0,1) < individual_‌resist_‌transmission_‌prob

then [ask **sexual partner** [*set resist_TAM ‘high’*]]

]

]

}

]

#On 1^st^ line ART

ask **people** with (HIV_‌status = ‘positive’ and ART_‌status = ‘on 1^st^ line ART’) [

for each **sexual partner** with (*HIV_‌status = ‘negative’*) **{**

if random-binomial(10, HIV_‌transmission_‌prob) > 0

then [

ask **sexual partner** [*set* *HIV_‌status ‘positive’*]

ask **sexual partner** [*set* *primary_‌infection ‘yes’*]

if resist_NNRTI ≠ ‘none’ and random-uniform [0,1) < resist_transmission_ART

then [

ask **sexual partner** [*set resist_NNRTI ‘medium’*]

if resist_NNRTI = ‘high’ and random-uniform [0,1) <

resist_transmission_ART

then [ask **sexual partner** [*set* *resist_NNRTI ‘high’*]]

]

if resist_NRTI ≠ ‘none’ and random-uniform [0,1) resist_transmission_ART * reduced_‌NRTI_‌resist_‌transmit

then [

ask **sexual partner** [*set resist_NRTI ‘medium’*]

if resist_NRTI = ‘high’ and random-uniform [0,1) <

resist_transmission_ART * reduced_‌NRTI_‌resist_‌trans

then [ask **sexual partner** [*set resist_NRTI ‘high’*]]

]

if resist_PI ≠ ‘none’ and random-uniform [0,1) < resist_transmission_other_ART

then [

ask **sexual partner** [*set resist_PI ‘medium’*]

if resist_PI = ‘high’ and random-uniform [0,1) < resist_transmission_other_ART

then [ask **sexual partner** [*set* *resist_PI ‘high’*]]

]

if resist_TAM≠ ‘none’ and random-uniform [0,1) < resist_transmission_ART

then [

ask **sexual partner** [*set resist_TAM ‘medium’*]

if resist_TAM= ‘high’ and random-uniform [0,1) < resist_transmission_ART

then [ask **sexual partner** [*set resist_TAM ‘high’*]]

]

]

}

]

#On 2^nd^ line ART

ask **people** with (HIV_‌status = ‘positive’ and ART_‌status = ‘on 2^nd^ line ART’) [

for each **sexual partner** with (*HIV_‌status = ‘negative’*) **{**

if random-binomial(10, HIV_‌transmission_‌prob) > 0

then [

ask **sexual partner** [*set* *HIV_‌status ‘positive’*]

ask **sexual partner** [*set* *primary_‌infection ‘yes’*]

if resist_NNRTI ≠ ‘none’ and random-uniform [0,1) < resist_transmission_other_ART

then [

ask **sexual partner** [*set resist_NNRTI ‘medium’*]

if resist_NNRTI = ‘high’ and random-uniform [0,1) <

resist_transmission_other_ART

then [ask **sexual partner** [*set* *resist_NNRTI ‘high’*]]

]

if resist_NRTI ≠ ‘none’ and random-uniform [0,1) resist_transmission_ART * reduced_‌NRTI_‌resist_‌transmit

then [

ask **sexual partner** [*set resist_NRTI ‘medium’*]

if resist_NRTI = ‘high’ and random-uniform [0,1) <

resist_transmission_ART * reduced_‌NRTI_‌resist_‌trans

then [ask **sexual partner** [*set resist_NRTI ‘high’*]]

]

if resist_PI ≠ ‘none’ and random-uniform [0,1) < resist_transmission_ART

then [

ask **sexual partner** [*set resist_PI ‘medium’*]

if resist_PI = ‘high’ and random-uniform [0,1) < resist_transmission_ART

then [ask **sexual partner** [*set* *resist_PI ‘high’*]]

]

if resist_TAM≠ ‘none’ and random-uniform [0,1) < resist_transmission_ART

then [

ask **sexual partner** [*set resist_TAM ‘medium’*]

if resist_TAM= ‘high’ and random-uniform [0,1) < resist_transmission_ART

then [ask **sexual partner** [*set resist_TAM ‘high’*]]

]

]

}

]

### Drop out care

#### Description

This submodel simulates people dropping out of pre-ART care in the model. Dropping out of ART is a separate submodel, described in the section ‘Drop out ART’. The rate of dropping out of care can be different in men and women, and is higher in people with poor health seeking behaviour/adherence. After dropping out of pre-ART care, people can re-enter care again through being tested for HIV and linked to care, as described in the section ‘HIV test’. Being in care reduces HIV mortality rates (see section ‘mortality’), and can increase the probability of starting ART each month (see section ‘start ART’).

#### Process

ask **(wo)men** with (care_status = ‘in care’ and ART_status = ‘not on ART’) [

if random-uniform [0,1) < care_‌dropout_‌multiplier * dropout_‌rateM/F / adherence_‌adjustment

then [set care_status ‘not in care’]

]

### HIV test

#### Description

HIV testing is divided up into two steps in the model: testing people who are HIV+ and testing people who are HIV-. Two parameters control the baseline rate of testing in men and women respectively: HIV_‌test_‌rate_‌M and HIV_‌test_‌rate_‌F. The values of these parameters can change over time. People can only test for HIV if at least six months had passed since their last HIV test. A third parameter allows for a higher rate of testing in HIV+ people: increased_‌test_‌rate_‌HIV+. Finally, people with poor health seeking behaviour/adherence have a lower rate of testing. If people are tested within one month of being infected with HIV, there is a 75% chance that the test will give a false negative result. Under these circumstances, their time_since_HIV_test parameter is not reset to zero.

Upon testing positive, men and women can be immediately linked to pre-ART care with probability link_‌to_‌care_‌M and link_‌to_‌care_‌F respectively. Probabilities of linking to care can be lower for people with poor health seeking behaviour/adherence. The probabilities of immediate linkage to care can change over time.

Finally, people who are linked to pre-ART care can start ART immediately if they have a CD4 count below the threshold for starting ART. It is assumed that the implementation of recent changes in CD4 threshold is/was slow (the change from 250-350 cells/µL in 2010 and from 350-500 cells/µL in 2014). In addition to the CD4 threshold, an effective CD4 threshold is therefore also simulated. This is set equal to 250 cells/µL in 2010 and 2011, 350 cells/µL in 2014 and 2015, and equal to the CD4 threshold in all other years. The probability of immediately starting ART can be lower for people with CD4 counts between the effective threshold and the threshold than for people with CD4 counts below the effective threshold.

#### Process

#Testing HIV positive people

ask **(wo)men** with (HIV_‌status = ‘positive’ and ART_‌status = ‘never’ and care_status = ‘not in care’ and time_since_‌HIV_‌test >= 6) [

ifelse time_since_infection = 0 and random-uniform [0,1) < 0.75

then [set ever_‌tested ‘yes’]

otherwise [

if random-uniform [0,1) < HIV_‌test_‌rate_‌M/F * increased_‌test_‌rate_‌HIV+ * adherence_‌adjustment [

set ever_‌tested ‘yes’ set time_‌since_‌HIV_‌test 0

#linkage to care

if random-uniform [0,1) < link_‌to_‌care_‌M/F * adherence_‌adjustment and year > 2002

then [

set care_status ‘in care’

#immediate ART start

if (random-uniform [0,1) < prob_‌immediate_‌ART_‌start and CD4_‌count < effective_‌CD4_‌threshold)

then [immediate_‌ART_‌start] #See section ‘Start ART’

if (random-uniform [0,1)< prob_‌immediate_‌ART_‌start * proportion_‌observing_‌new_‌thresholds and CD4_‌count < CD4_‌threshold and CD4_‌count > effective_‌CD4_‌threshold)

then [immediate_‌ART_‌start] #See section ‘Start ART’

]

]

]

#Testing HIV negative people

ask **(wo)men** with (HIV_‌status = ‘negative’ and time_‌since_‌HIV_‌test >= 6) [

if (random-uniform [0,1) <HIV_‌test_‌rate_‌M/F * adherence_‌adjustment

then [set ever_tested ‘yes’ set time_‌since_‌HIV_‌test 0]

]

### Track ART

#### Description

This submodel tracks the duration of time people have left with primary infections, moves people from primary to post-primary infections, and tracks the decline in CD4 count for people who are HIV positive and not on ART.

#### Process

ask **people** with (primary_‌infection = ‘yes’) [

if primary_‌time < 0

then [

set primary_‌infection ‘no’

set primary_‌time (primary_‌time – 1)

]

]

ask **people** with (HIV_‌status = ‘positive’ and ‌ART_status = ‘not on ART’) [

ifelse CD4_‌count > 0

then [set CD4_‌count ((√CD4_‌count – CD4_‌decline_‌rate) ^ 2)]

otherwise [set CD4_‌count 0]

]

### Restart ART

#### Description

This submodel restarts people onto ART who have previously dropped out of ART. Rates of restarting can be different for men and women, and are lower for people with poor health seeking behaviour/adherence.

#### Process

ask **(wo)men** with (ART_‌status = ‘dropped out of 1^st^ line’) [

if random-uniform [0,1) < restart_‌rateM/F * adherence_‌adjustment

then [

set ‌ART_status ‘1^st^ line ≤1 year’

set time_‌on_‌ART 0

set care_status ‘in care’

]

ask **(wo)men** with (ART_‌status = ‘dropped out of 2^nd^ line’) [

if random-uniform [0,1) < restart_‌rateM/F * adherence_‌adjustment

then [

set ‌ART_status ‘2^nd^ line ≤1 year’

set time_‌on_‌ART 0

set care_status ‘in care’

]

]

### Drop out ART

#### Description

This submodel is responsible for people dropping out of ART. Rates of dropping out can be different for men and women, are higher for people with poor health seeking behaviour/adherence, and are higher during the first year on an ART regimen. People who drop out of ART in the model are assumed to drop out of HIV care entirely. The drop out stages in the model are designed to represent people who have ceased taking ART for a period of three months or more. Treatment pauses of less than three months duration are not modelled explicitly.

Changes in CD4 count while on ART are not explicitly simulated, as current CD4 count is not used to determine any characteristics/probabilities for a person while they are on ART. CD4 count once again becomes important in the model when someone drops out of ART. To allow for changes in CD4 count that may have occurred while a person was on ART, step changes in CD4 count upon dropping out of ART can be simulated in the model. The size and direction of the step changes can be different for people dropping out of ART after less than and more than one year on ART. People’s CD4 count upon dropping out cannot exceed a model-wide threshold value (max_‌ART_‌CD4)

#### Process

ask **(wo)men** with (ART_‌status = ‘on_‌ART ≤1 year’ and time_‌on_‌ART > 2) [

if random-uniform [0,1) < dropout_‌rateM/F * increase_‌dropout_‌y1 / adherence_‌adjustment

then [

set ART_‌status ‘dropped out ART’

#People dropping out of 1^st^ line ART set their ART_‌status to ‘dropped out of 1^st^ line’. People dropping out of 2^nd^ line ART set their ART_‌status to ‘dropped out of 2^nd^ line’.

set care_status ‘not in care’

ifelse (CD4_‌count + increase_‌CD4_‌dropout_‌y1) < max_‌ART_‌CD4

then [set CD4_‌count (CD4_‌count + increase_‌CD4_‌dropout_‌y1)]

otherwise [set CD4_‌count max_‌ART_‌CD4]

if CD4_‌count < 0

then [set CD4_‌count 0]

]

]

ask **(wo)men** with (ART_‌status = ‘on_‌ART >1 year’) [

if random-uniform [0,1) < dropout_‌rateM/F / adherence_‌adjustment

then [set ART_‌status ‘dropped out ART’

#People dropping out of 1^st^ line ART set their ART_‌status to ‘dropped out of 1^st^ line’. People dropping out of 2^nd^ line ART set their ART_‌status to ‘dropped out of 2^nd^ line’.

set care_status ‘not in care’

ifelse (CD4_‌count + increase_‌CD4_‌dropout_‌y2) < max_‌ART_‌CD4

then [set CD4_‌count (CD4_‌count + increase_‌CD4_‌dropout_‌y2)]

otherwise [set CD4_‌count max_‌ART_‌CD4]

if CD4_‌count < 0

then [set CD4_‌count 0]

]

]

### Start ART

#### Description

There are five ways of starting ART:

1. Immediately after testing positive
2. Due to clinical events (when not in pre-ART care)
3. Due to clinical events (when in pre-ART care)
4. From pre-ART care when below the CD4 threshold
5. Due to option B+ (women only, from 2014)

#### Process

1. #this section of the submodel is only executed when called by individual people within other sections of the code (See section HIV test). For this reason, although it can only be executed by people, it does not start ‘Ask…’

set time_‌on_‌ART 0

set ART_‌status ‘1^st^ line ≤1 year’

if random-uniform [0,1) < prob_‌low_‌adherence_‌if_‌no_‌event

then [set adherence_‌group ‘low’]

1. ask **people** with (HIV_‌status = ‘positive’ and ART_‌status = ‘never’ and care_status = ‘not in care’) [

if random-uniform [0,1) < (CD4_‌stage3or4_‌multiplier * adherence_‌adjustment * ((0.0193 / exp(350 * CD4_‌stage3or4_‌slope)) * exp(CD4_‌stage3or4_‌slope * CD4_‌count)))

then [

set time_‌on_‌ART 0

set ART_‌status ‘1^st^ line ≤1 year’

set care_status ‘in care’

set ever_tested ‘yes’

]

]

1. ask **people** with (HIV_‌status = ‘positive’ and ART_‌status = ‘never’ and care_status = ‘in care’) [

if random-uniform [0,1) < (CD4_‌in_‌care_‌stage3or4_‌multiplier * ((0.0193 / exp(350 * CD4_‌stage3or4_‌slope)) * exp(CD4_‌stage3or4_‌slope * CD4_‌count)))

then [

set time_‌on_‌ART 0

set ART_‌status ‘1^st^ line ≤1 year’

]

]

1. ask **people** with (HIV_‌status = ‘positive’ and ART_‌status = ‘never’ and care_status = ‘in care’ and time_since_CD4_test >= 6) [

if random-uniform [0,1) < CD4_test_rate_in_care

then [

set time_since_CD4_test 0

if CD4_‌count < effective_‌CD4_‌threshold

then [

set time_‌on_‌ART 0

set ART_‌status ‘1^st^ line ≤1 year’

if random-uniform [0,1) < prob_‌low_‌adherence_‌if_‌no_‌event

then [set adherence_‌group ‘low’]

]

if CD4_‌count > effective_‌CD4_‌threshold and CD4_‌count < CD4_‌threshold

then [

set time_‌on_‌ART 0

set ART_‌status ‘1^st^ line ≤1 year’

if random-uniform [0,1) < prob_‌low_‌adherence_‌if_‌no_‌event

then [set adherence_‌group ‘low’]

]

]

1. ask **women** with (HIV_‌status = ‘positive’ and ART_‌status = ‘never’) [

if random-uniform [0,1) < pregnancy_‌rate * B+_‌coverage * adherence_‌adjustment

then [

set time_‌on_‌ART 0

set ART_‌status ‘1^st^ line ≤1 year’

if random-uniform [0,1) < prob_‌low_‌adherence_‌if_‌no_‌event

then [set adherence_‌group ‘low’]

]

]

### Switch to 2nd line

#### Description

People in the model can switch to 2^nd^ line ART after they have spent a year or more on ART continuously. Anyone in the model on 1^st^ line ART for more than one year with less than the maximum number of fully active drugs can switch to 2^nd^ line ART, with the probability of switching increasing linearly as the number of active drugs falls from the maximum to zero. This is referred to as ‘necessary switching’. Low adherence people on ART for more than one year continuously can also switch to 2^nd^ line ART due to ‘unnecessary switching’. The rate at which this occurs is constant, and independent of the level of drug resistance.

#### Process

ask **people** with (ART_‌status = ‘1^st^ line >1 year’) [

if random-uniform [0,1) < max_‌necessary_‌switch_‌rate * (1 - resist_‌ART)

then [

set ART_‌status = ‘2^nd^ line ≤1 year’

set time_‌on_‌ART 0

]

]

ask **people** with (ART_‌status = ‘1^st^ line >1 year’ and adherence_‌group = ‘low’) [

if random-uniform [0,1) < prob_‌unnecessary_‌switch

then [

set ART_‌status = ‘2^nd^ line ≤1 year’

set time_‌on_‌ART 0

]

]

### Develop resistance

#### Description

If someone is on an ART regimen containing a drug that selects for a particular type of resistance, then they can develop that type of resistance. There are four types of drug resistance in the model: major NNRTI mutations, major NRTI mutations, major PI mutations, and TAMs. People can have no, medium, or high resistance of each type. There is a baseline rate of acquiring drug resistance (acquire_‌resist_‌rate_‌baseline), common to all drugs and individuals in the model, and to both ART regimens. Rates of acquiring drug resistance are higher in people with low adherence, and increase as the number of active drugs an individual is on declines. Rates are lower if someone is on a regimen containing a boosted protease inhibitor (i.e. 2^nd^ line ART). In general, resistance can only change from no resistance, to medium resistance, to high resistance. However, due to the large reductions in drug activity caused by certain common NNRTI mutations, there is a probability that someone who develops NNRTI resistance will immediately develop a high level of resistance (prob_‌full_‌NNRTI_‌resist).

#### Process

ask **people** with (ART_‌status = ‘on ART’) [

Update_‌resist_ART

]

#People on 1^st^ line, developing major NNTRI mutations

ask **people** with (ART_‌status = ‘on 1^st^ line’ and adherence_‌group = ‘high’ and resist_‌NNRTI = ‘medium’) [

if random-uniform [0,1) < acquire_‌resist_‌rate_‌baseline * resist_‌gain_‌current_‌resist

then [set resist_‌NNRTI ‘high’]

]

ask **people** with (ART_‌status = ‘on 1^st^ line’ and adherence_‌group = ‘low’ and resist_‌NNRTI = ‘medium’) [

if random-uniform [0,1) < acquire_‌resist_‌rate_‌baseline * acquire_‌resist_‌increase_‌low_‌adherence * resist_‌gain_‌current_‌resist

then [set resist_‌NNRTI ‘high’]

]

ask **people** with (ART_‌status = ‘on 1^st^ line’ and adherence_‌group = ‘high’ and resist_‌NNRTI = ‘low’) [

if random-uniform [0,1) < acquire_‌resist_‌rate_‌baseline * resist_‌gain_‌current_‌resist

then [

ifelse random-float 1 < prob_‌full_‌NNRTI_‌resist

then [set resist_‌NNRTI ‘high’]

otherwise [set resist_‌NNRTI ‘medium’]

]

]

ask **people** with (ART_‌status = ‘on 1^st^ line’ and adherence_‌group = ‘low’ and resist_‌NNRTI = ‘low’) [

if random-uniform [0,1) < acquire_‌resist_‌rate_‌baseline * acquire_‌resist_‌increase_‌low_‌adherence * resist_‌gain_‌current_‌resist

then [

if random-float 1 < prob_‌full_‌NNRTI_‌resist

then [set resist_‌NNRTI ‘high’]

otherwise [set resist_‌NNRTI ‘medium’]

]

]

#People on 1^st^ line, developing major NTRI mutations

ask **people** with (ART_‌status = ‘on 1^st^ line’ and adherence_‌group = ‘high’ and resist_‌NRTI = ‘medium’)

if random-uniform [0,1) < acquire_‌resist_‌rate_‌baseline * resist_‌gain_‌current_‌resist

then [set resist_‌NRTI ‘high’]

]

ask **people** with (ART_‌status = ‘on 1^st^ line’ and adherence_‌group = ‘low’ and resist_‌NRTI = ‘medium’)

if random-uniform [0,1) < acquire_‌resist_‌rate_‌baseline * acquire_‌resist_‌increase_‌low_‌adherence * resist_‌gain_‌current_‌resist

then [set resist_‌NRTI ‘high’]

]

ask **people** with (ART_‌status = ‘on 1^st^ line’ and adherence_‌group = ‘high’ and resist_‌NRTI = ‘low’)

if random-uniform [0,1) < acquire_‌resist_‌rate_‌baseline * resist_‌gain_‌current_‌resist

then [set resist_‌NRTI ‘medium’]

]

ask **people** with (ART_‌status = ‘on 1^st^ line’ and adherence_‌group = ‘low’ and resist_‌NRTI = ‘low’)

if random-uniform [0,1) < acquire_‌resist_‌rate_‌baseline * acquire_‌resist_‌increase_‌low_‌adherence * resist_‌gain_‌current_‌resist

then [set resist_‌NRTI ‘medium’]

]

#People on 2^nd^ line, developing major NTRI mutations

ask **people** with (ART_‌status = ‘on 2^nd^ line’ and adherence_‌group = ‘high’ and resist_‌NRTI = ‘medium’)

if random-uniform [0,1) < acquire_‌resist_‌rate_‌baseline * resist_‌gain_‌current_‌resist * reduced_‌acquire_‌resist_‌rate_‌if_‌PI_‌present

then [set resist_‌NRTI ‘high’]

]

ask **people** with( ART_‌status = ‘on 2^nd^ line’ and adherence_‌group = ‘low’ and resist_‌NRTI = ‘medium’)

if random-uniform [0,1) < acquire_‌resist_‌rate_‌baseline * acquire_‌resist_‌increase_‌low_‌adherence * resist_‌gain_‌current_‌resist * reduced_‌acquire_‌resist_‌rate_‌if_‌PI_‌present

then [set resist_‌NRTI ‘high’]

]

ask **people** with (ART_‌status = ‘on 2^nd^ line’ and adherence_‌group = ‘high’ and resist_‌NRTI = ‘low’)

if random-uniform [0,1) < acquire_‌resist_‌rate_‌baseline * resist_‌gain_‌current_‌resist * reduced_‌acquire_‌resist_‌rate_‌if_‌PI_‌present

then [set resist_‌NRTI ‘medium’]

]

ask **people** with (ART_‌status = ‘on 2^nd^ line’ and adherence_‌group = ‘low’ and resist_‌NRTI = ‘low’)

if random-uniform [0,1) < acquire_‌resist_‌rate_‌baseline * acquire_‌resist_‌increase_‌low_‌adherence * resist_‌gain_‌current_‌resist * reduced_‌acquire_‌resist_‌rate_‌if_‌PI_‌present

then [set resist_‌NRTI ‘medium’]

]

#People on 2^nd^ line, developing TAMs

ask **people** with (ART_‌status = ‘on 2^nd^ line’ and adherence_‌group = ‘high’ and resist_‌TAM = ‘medium’)

if random-uniform [0,1) < acquire_‌resist_‌rate_‌baseline * resist_‌gain_‌current_‌resist * reduced_‌acquire_‌resist_‌rate_‌if_‌PI_‌present

then [set resist_‌TAM ‘high’]

]

ask **people** with (ART_‌status = ‘on 2^nd^ line’ and adherence_‌group = ‘low’ and resist_‌TAM = ‘medium’)

if random-uniform [0,1) < acquire_‌resist_‌rate_‌baseline * acquire_‌resist_‌increase_‌low_‌adherence * resist_‌gain_‌current_‌resist * reduced_‌acquire_‌resist_‌rate_‌if_‌PI_‌present

then [set resist_‌TAM ‘high’]

]

ask **people** with (ART_‌status = ‘on 2^nd^ line’ and adherence_‌group = ‘high’ and resist_‌TAM = ‘low’)

if random-uniform [0,1) < acquire_‌resist_‌rate_‌baseline * resist_‌gain_‌current_‌resist * reduced_‌acquire_‌resist_‌rate_‌if_‌PI_‌present

then [set resist_‌TAM ‘medium’]

]

ask **people** with (ART_‌status = ‘on 2^nd^ line’ and adherence_‌group = ‘low’ and resist_‌TAM = ‘low’)

if random-uniform [0,1) < acquire_‌resist_‌rate_‌baseline * acquire_‌resist_‌increase_‌low_‌adherence * resist_‌gain_‌current_‌resist * reduced_‌acquire_‌resist_‌rate_‌if_‌PI_‌present

then [set resist_‌TAM ‘medium’]

]

#People on 2^nd^ line, developing major PI mutations

ask **people** with (ART_‌status = ‘on 2^nd^ line’ and adherence_‌group = ‘high’ and resist_‌PI = ‘medium’)

if random-uniform [0,1) < acquire_‌resist_‌rate_‌baseline * resist_‌gain_‌current_‌resist * reduced_‌acquire_‌resist_‌rate_‌if_‌PI_‌present

then [set resist_‌PI ‘high’]

]

ask **people** with (ART_‌status = ‘on 2^nd^ line’ and adherence_‌group = ‘low’ and resist_‌PI = ‘medium’)

if random-uniform [0,1) < acquire_‌resist_‌rate_‌baseline * acquire_‌resist_‌increase_‌low_‌adherence * resist_‌gain_‌current_‌resist * reduced_‌acquire_‌resist_‌rate_‌if_‌PI_‌present

then [set resist_‌PI ‘high’]

]

ask **people** with (ART_‌status = ‘on 2^nd^ line’ and adherence_‌group = ‘high’ and resist_‌PI = ‘low’)

if random-uniform [0,1) < acquire_‌resist_‌rate_‌baseline * resist_‌gain_‌current_‌resist * reduced_‌acquire_‌resist_‌rate_‌if_‌PI_‌present

then [set resist_‌PI ‘medium’]

]

ask **people** with (ART_‌status = ‘on 2^nd^ line’ and adherence_‌group = ‘low’ and resist_‌PI = ‘low’)

if random-uniform [0,1) < acquire_‌resist_‌rate_‌baseline * acquire_‌resist_‌increase_‌low_‌adherence * resist_‌gain_‌current_‌resist * reduced_‌acquire_‌resist_‌rate_‌if_‌PI_‌present

then [set resist_‌PI ‘medium’]

]

### Annual output

#### Description

Each model year, a large number of summary statistics are outputted, giving details of the number of people with different characteristics at the end of a year (e.g. number of women with primary stage infections), the number of person months with certain characteristics that have occurred that year (e.g. total person months on ART), the number of times people’s characteristics have changed in certain ways (e.g. number of males newly infected with HIV by women who have dropped out of ART), or median times between events (e.g. median years between HIV infection and death). The details of how these outputs are tracked in the model are not given in the model description, as they would greatly increase its complexity. The tracking and production of outputs have no effect on the model results.

### Change year

#### Description

By this point in the schedule, all submodels which affect people in the model have run. This and subsequent submodels make changes to model parameters in preparation for the start of the next model month. This submodel changes the year every 12 months.

#### Process

set year (year + 1)

### Scale up ART

#### Description

The model contains a number of model wide parameters which have different values at different points in time. The values are changed in this submodel (parameters related to ART scale-up), and in the submodel ‘change risk behaviour’ below (parameters related to risk behaviour).

#### Process

#Change HIV testing rates

if year = 2005 then [set HIV_‌test_‌rate_‌M HIV_‌test_‌rate_‌M_‌2005 set HIV_‌test_‌rate_‌F HIV_‌test_‌rate_‌F_‌2005]

if year = 2007 then [set HIV_‌test_‌rate_‌M HIV_‌test_‌rate_‌M_‌2007 set HIV_‌test_‌rate_‌F HIV_‌test_‌rate_‌F_‌2007]

if year = 2012 then [set HIV_‌test_‌rate_‌M HIV_‌test_‌rate_‌M_‌2012 set HIV_‌test_‌rate_‌F HIV_‌test_‌rate_‌F_‌2012]

#Change linkage to HIV care and starting ART rates

if year = 2008

then [

set link_‌to_‌care_‌F (link_‌to_‌care_‌F_2008)

set link_‌to_‌care_‌M (link_‌to_‌care_‌F_2008 * reduced_link_‌to_‌care_‌M)

set CD4_‌stage3or4_‌multiplier CD4_‌stage3or4_‌multiplier_2008

set CD4_‌in_‌care_‌stage3or4_‌multiplier (CD4_‌stage3or4_‌multiplier_2008

* increased_CD4_stage3or4_multiplier_in_care)

set CD4_test_rate_in_care ‌CD4_test_rate_in_care_‌2008

set prob_‌immediate_‌ART_‌start ‌prob_‌immediate_‌ART_‌start_‌2008

]

if year = 2012

then [

set link_‌to_‌care_‌F (link_‌to_‌care_‌F_2012)

set link_‌to_‌care_‌M (link_‌to_‌care_‌F_2012 * reduced_link_‌to_‌care_‌M)

set CD4_‌stage3or4_‌multiplier CD4_‌stage3or4_‌multiplier_2012

set CD4_‌in_‌care_‌stage3or4_‌multiplier (CD4_‌stage3or4_‌multiplier_2012

* increased_CD4_stage3or4_multiplier_in_care)

set CD4_test_rate_in_care ‌CD4_test_rate_in_care_‌2012

set prob_‌immediate_‌ART_‌start prob_‌immediate_‌ART_‌start_‌2012

]

#Change the CD4 threshold for starting ART

if year = 2003 then [set CD4_‌threshold 200 set effective_‌CD4_‌threshold 200]

if year = 2009 then [set CD4_‌threshold 250 set effective_‌CD4_‌threshold 250]

if year = 2010 then [set CD4_‌threshold 350]

if year = 2012 then [set effective_‌CD4_‌threshold 350]

if year = 2014 then [set CD4_‌threshold 500]

if year = 2016 then [set effective_‌CD4_‌threshold 500]

### Change risk behaviour

#### Description

Two step changes in sexual risk behaviour are modelled, the 1^st^ in 1992, and the 2^nd^ in 2004.

#### Process

if year = 1992

then [

set high_‌risk_‌contact_‌rateM high_‌risk_‌contact_‌rateM_‌2

set low_‌risk_‌contact_‌rateM low_‌risk_‌contact_‌rateM_‌2

#This changes the value of the concurrency parameter for people who already exist

ask **(wo)men** with concurrency_‌parameter = high_‌concurrency_‌parameterM/F [

set concurrency_‌parameter high_‌concurrency_‌parameterM/F_‌2

recalculate_‌availability

]

#This changes the values of the concurrency parameters assigned to each concurrency group for people created in future months

set high_‌concurrency_‌parameterF high_‌concurrency_‌parameterF_‌2

set high_‌concurrency_‌parameterM high_‌concurrency_‌parameterM_‌2

]

if year = 2004

then [

set high_‌risk_‌contact_‌rateM high_‌risk_‌contact_‌rateM_‌3

set low_‌risk_‌contact_‌rateM low_‌risk_‌contact_‌rateM_‌3

#This changes the value of the concurrency parameter for people who already exist

ask **(wo)men** with concurrency_‌parameter = high_‌concurrency_‌parameterM/F [

set concurrency_‌parameter high_‌concurrency_‌parameterM/F_‌3

recalculate_‌availability

]

#This changes the values of the concurrency parameters assigned to each concurrency group for people created in future months

set high_‌concurrency_‌parameterF high_‌concurrency_‌parameterF_‌3

set high_‌concurrency_‌parameterM high_‌concurrency_‌parameterM_‌3

]

### Seed HIV

#### Description

HIV is seeded into the model in 1970 by changing the HIV status of a random selection of people to positive. The probability of being chosen as a seed is different for people with low and high risk sexual behaviour.

#### Process

if year = 1970

then [ask **people** with (risk = ‘high’)

if random-uniform [0,1) < 0.1

then [set HIV_‌status ‘positive’]

]

ask **people** with (risk = ‘low’)

if random-uniform [0,1) < 0.05

then [set HIV_‌status ‘positive’]

]

### Seed resistance

#### Description

HIV drug resistance is seeded into the model in 2003 by changing the level of resistance to NNRTI class drugs of a proportion of all HIV+ people (chosen at random) to medium.

#### Process

if year = 2003

then [ask **people** with (HIV_‌status = ‘positive’)

if random-uniform [0,1) < seed_resist_2003

then [set resist_‌NNRTI ‘medium’]

]

### Count women

#### Description

As people enter into the model at age 15 years, the number of new people added to the model each month is set to be proportional to the number of women in the model 15 years previously. This is done by tracking the number of women in the model at the end of each year using a list containing 15 items. At the end of each year, the parameter women_‌15y_‌ago (which is used to determine the number of new people the next year) is set to be equal to the 1^st^ item in the list. That item is then deleted, and the current number of women in the model added to the end of the list.

#### Process

set women_‌15y_‌ago (1^st^ item from women_‌15y_‌ago_‌list)

set women_‌15y_‌ago_‌list (remove 1^st^ item from women_‌15y_‌ago_‌list)

add (count **women**) to end of women_‌15y_‌ago_‌list

## Submodels called from within submodels

### Recalculate_‌availability

#### Description

The probability that someone is available to start additional partnerships is a function of their concurrency parameter and the number of sexual partners that they currently have. With a concurrency parameter of 0, people cannot form more than sexual partnership at a time (serial monogamy). With a concurrency parameter of 1, people are always available to start new partnerships, regardless of their current number of sexual partners. With intermediate values, the probability that someone is available to start a new partnership decreases as their number of current partners increases.

This submodel is called whenever new partnerships are formed (submodel ‘update partnerships’), and whenever partnerships end (submodels ‘update partnerships’ and ‘mortality’).

#### Process

#this section of the model is only executed when called by individual people within other sections of the code. For this reason, although it can only be executed by people, it does not start ‘Ask…’

ifelse random-uniform [0,1) < (concurrency_‌parameter ^ (count **partners**))

then [set available ‘yes’]

otherwise [set available ‘no’]

### Update_‌resist_‌increased_‌mortality

#### Description

Mortality rates for people on ART increase as their level of resistance to the drug regimen that they are on increases. With 100% resistance, their mortality rate is the same as it would be if they were in care but not on ART.

#### Process

update_‌resist_‌ART

for ***‘i’*** in (1, 2, 3, 4, 5, 6, 7, 8, 9) {

if ART_‌status = ‘ART ≤1 year’ and CD4_‌group = ***‘i’***

then [set resist_‌increased_‌mortality (exp((1 - resist_‌ART) * ln(HIV_‌mortality_‌rate_‌***’i’***/ (ARTy1_‌mortality_‌rate_‌***’i’*** *** reduced_‌mortality_‌in_‌care))))]

if ART_‌status = ‘ART >1 year’ and CD4_‌group = ***‘i’***

then [set resist_‌increased_‌mortality (exp((1 - resist_‌ART) * ln(HIV_‌mortality_‌rate_‌***’i’***/ (ARTy2_‌mortality_‌rate_‌***’i’*** *** reduced_‌mortality_‌in_‌care))))]

}

### Update_‌resist_‌increased_‌transmission

#### Description

Transmission probabilities for people on ART in the model increase as their level of resistance to the drug regimen that they are on increases. With 100% resistance, their transmission probability is the same as it would be if they were not on ART.

#### Process

update_‌resist_‌ART

for ***‘i’*** in (1, 2, 3, 4, 5, 6, 7, 8, 9) {

if CD4_‌group = ***‘i’***

then [set resist_‌increased_‌transmit (exp((1 - resist_‌ART) * ln(HIV_‌transmission_‌prob_‌***’i'***/ onART_‌HIV_‌transmission_‌prob)))]

}

### Update_‌resist_‌ART

#### Description

This submodel determines the overall level of resistance a person has to the drug regimen that they are on, based on their level of resistance to the three individual drugs they are taking.

#### Process

if ART_‌status = ‘on 1^st^ line’

then [

set resist_‌TDF min(resist_‌NRTI, resist_‌TAM)] #the level of resistance to TDF is assumed to be equal to the highest level of resistance due to major NRTI mutations or due to TAMs.

set resist_‌ART (resist_‌NNRTI + resist_‌NRTI * NRTI_‌weight + resist_‌TDF * NRTI_‌weight) / max_‌total_‌ART

]

if ART_‌status = ‘on 2^nd^ line’

then [set resist_‌ART (resist_‌PI * PI_‌weight + resist_‌NRTI * NRTI_‌weight + resist_‌TAM * NRTI_‌weight) / max_total_‌ART]

ifelse (resist_‌NNRTI + resist_‌NRTI + resist_‌TDF + resist_‌PI) = 4

then [set any_‌resistance ‘no’]

otherwise [set any_‌resistance ‘yes]

set resist_‌gain_‌current_‌resist (-3 * current_‌resist_‌resist_‌gain_‌link * resist_‌ART + 3 * current_‌resist_‌resist_‌gain_‌link + 3 * resist_‌ART - 2)

### Calculate_‌CD4_‌group

#### Description

CD4 count is explicitly tracked as a continuous variable for all HIV+ people in the model. For purposes of determining certain rates and probabilities (e.g. HIV mortality rates), CD4 count is also grouped into a categorical variable. Primary infections override CD4 count, and people with a primary infection are placed into the primary infection category, regardless of their CD4 count.

#### Process

ifelse primary_‌infection = ‘yes’

then [set CD4_‌group 1]

otherwise [

if CD4_‌count > 500 then [set CD4_‌group 2]

if CD4_‌count > 350 and CD4_‌count ≤ 500 then [set CD4_‌group 3]

if CD4_‌count > 250 and CD4_‌count ≤ 350 then [set CD4_‌group 4]

if CD4_‌count > 200 and CD4_‌count ≤ 250 then [set CD4_‌group 5]

if CD4_‌count > 150 and CD4_‌count ≤ 200 then [set CD4_‌group 6]

if CD4_‌count > 100 and CD4_‌count ≤ 150 then [set CD4_‌group 7]

if CD4_‌count > 50 and CD4_‌count ≤ 100 then [set CD4_‌group 8]

if CD4_‌count ≤ 50 then [set CD4_‌group 9]

]

## Interventions

#### Description

Interventions were implemented in the model at the start of 2016, and the intervention submodel was called at the start of 2016, 2017, and 2018. Six single intervention components were simulated:

1. Increased HIV testing.
   1. The probability of being tested for HIV each month was doubled. This excluded testing due to pregnancy, and testing that occured when people not in care started ART due to stage 3 or 4 conditions.
2. No CD4 threshold.
   1. The CD4 threshold was removed in 2016, and all clinics were assumed to have adopted the new guidelines by 2018. It was not assumed that all people in pre-ART care or all people newly linked to care would immediately start ART. Instead, the rate of starting ART from pre-ART care when below the CD4 threshold was increased by 50%, and the probability that people newly linked to care would immediately start ART was increased by 50%.
3. Improved retention.
   1. The rate of dropping out of ART was halved
4. Increased restart.
   1. The rate of restarting ART after dropping out was doubled.
5. Improved linkage to care.
   1. The probability of being linked to care following a positive HIV test was doubled.
6. Improved pre-ART care.
   1. The rate of dropping out of pre-ART care was halved.
   2. The probability of linking to care following a positive HIV test was doubled.
   3. The rate of starting ART from pre-ART care when eligible was doubled.
   4. The probability of starting ART following a clinical event while in pre-ART care was doubled.

Thirteen combinations of two single intervention components were also simulated, consisting of all plausible combinations. Two possible combinations were considered implausible and were not simulated: no CD4 threshold for ART initiation combined with improved pre-ART care (no CD4 threshold would lead to the phasing out of widespread pre-ART care) and improved linkage to care combined with improved pre-ART care (the improved pre-ART care intervention includes improved linkage to care).

Finally, two intensive, multi-component interventions were simulated: Universal test and treat (UTT; combines components 1,2, and 5) and Universal, test, treat, and keep (UTTK; combines components 1-5).

No changes were made to the model in the baseline scenario.

#### Process

**Increased HIV testing:**

if year = 2016 then [set HIV_test_rate_M/F (2 * HIV_test_rate_M/F)]

**No CD4 threshold for ART initiation:**

if year = 2016

then [

set CD4_‌threshold 999999 set effective_‌CD4_‌threshold 500

set prob_immediate_ART_start (prob_immediate_ART_start * 1.5)

set CD4_test_rate_in_care (CD4_test_rate_in_care * 1.5)

]

if year = 2018 then [set CD4_‌threshold 999999 set effective_‌CD4_‌threshold 999999]

**Improved retention on ART:**

if year = 2016 then [set dropout_‌rateM/F (dropout_‌rateM/F / 2)]

**Improved ART restart rates:**

if year = 2016 then [set restart_‌rateM/F (restart_‌rateM/F / 2)]

**Improved linkage to care:**

if year = 2016

then [

set link_to_care_M/F (link_to_care_M/F * 2)

]

**Improved pre-ART care:**

if year = 2016

then [

set care_‌dropout_‌multiplier (care_‌dropout_‌multiplier / 2)

set CD4_test_rate_in_care (CD4_test_rate_in_care * 2)

set link_to_care_M/F (link_to_care_M/F * 2)

set CD4_‌in_‌care_‌stage3or4_‌multiplier (CD4_‌in_‌care_‌stage3or4_‌multiplier * 2)

]

1. DeSA U: **World population prospects: The 2012 revision**. *Population Division of the Department of Economic and Social Affairs of the United Nations Secretariat, New York* 2013.

2. Mermin J, Were W, Ekwaru JP, Moore D, Downing R, Behumbiize P, Lule JR, Coutinho A, Tappero J, Bunnell R: **Mortality in HIV-infected Ugandan adults receiving antiretroviral treatment and survival of their HIV-uninfected children: a prospective cohort study**. *The Lancet* 2008, **371**(9614):752-759.

3. STD/AIDS Control Programme MoH: **Status of Antiretroviral Therapy Services in Uganda: Semi-Annual ART Report for January – June 2014**. In*.* Kampala, Uganda; 2014.

4. STD/AIDS Control Programme MoH: **Status of Antiretroviral Therapy Service Delivery in Uganda Quarterly Report for March – June 2010**. In*.* Kampala, Uganda; 2010.

5. Mills EJ, Bakanda C, Birungi J, Mwesigwa R, Chan K, Ford N, Hogg RS, Cooper C: **Mortality by baseline CD4 cell count among HIV patients initiating antiretroviral therapy: evidence from a large cohort in Uganda**. *Aids* 2011, **25**(6):851-855.

6. Anglaret X, Minga A, Gabillard D, Ouassa T, Messou E, Morris B, Traore M, Coulibaly A, Freedberg KA, Lewden C: **AIDS and non-AIDS morbidity and mortality across the spectrum of CD4 cell counts in HIV-infected adults before starting antiretroviral therapy in Cote d’Ivoire**. *Clinical infectious diseases* 2012, **54**(5):714-723.

7. Kranzer K, Lewis JJ, Ford N, Zeinecker J, Orrell C, Lawn SD, Bekker L-G, Wood R: **Treatment interruption in a primary care antiretroviral therapy programme in South Africa: cohort analysis of trends and risk factors**. *Journal of acquired immune deficiency syndromes (1999)* 2010, **55**(3):e17.

8. Hamers RL, Schuurman R, Sigaloff KC, Wallis CL, Kityo C, Siwale M, Mandaliya K, Ive P, Botes ME, Wellington M: **Effect of pretreatment HIV-1 drug resistance on immunological, virological, and drug-resistance outcomes of first-line antiretroviral treatment in sub-Saharan Africa: a multicentre cohort study**. *The Lancet infectious diseases* 2012, **12**(4):307-317.

9. Stadeli KM, Richman DD: **Rates of emergence of HIV drug resistance in resource-limited settings: a systematic review**. *Antiviral therapy* 2013, **18**(1):115.

10. Gupta R, Hill A, Sawyer AW, Pillay D: **Emergence of drug resistance in HIV type 1-infected patients after receipt of first-line highly active antiretroviral therapy: a systematic review of clinical trials**. *Clinical infectious diseases* 2008, **47**(5):712-722.

11. Von Wyl V, Yerly S, Böni J, Bürgisser P, Klimkait T, Battegay M, Furrer H, Telenti A, Hirschel B, Vernazza PL: **Emergence of HIV-1 drug resistance in previously untreated patients initiating combination antiretroviral treatment: a comparison of different regimen types**. *Archives of internal medicine* 2007, **167**(16):1782-1790.

12. Jain V, Sucupira MC, Bacchetti P, Hartogensis W, Diaz RS, Kallas EG, Janini LM, Liegler T, Pilcher CD, Grant RM: **Differential persistence of transmitted HIV-1 drug resistance mutation classes**. *Journal of Infectious Diseases* 2011, **203**(8):1174-1181.

13. Nicolosi A, Leite MLC, Musicco M, Arid C, Gavazzeni G, Lazzarin A: **The Efficiency of Male-to Female and Female-to-Male Sexual Transmission of the Human Immunodeficiency Virus: A Study of 730 Stable Couples**. *Epidemiology* 1994, **5**(6):570-575.

14. Baggaley RF, White RG, Hollingsworth TD, Boily M-C: **Heterosexual HIV-1 infectiousness and antiretroviral use: systematic review of prospective studies of discordant couples**. *Epidemiology* 2013, **24**(1):110-121.

15. Bellan SE, Dushoff J, Galvani AP, Meyers LA: **Reassessment of HIV-1 acute phase infectivity: accounting for heterogeneity and study design with simulated cohorts**. *PloS Med* 2015, **12**(3):e1001801.

16. Donnell D, Baeten JM, Kiarie J, Thomas KK, Stevens W, Cohen CR, McIntyre J, Lingappa JR, Celum C, Team PiPHHTS: **Heterosexual HIV-1 transmission after initiation of antiretroviral therapy: a prospective cohort analysis**. *The Lancet* 2010, **375**(9731):2092-2098.

17. Kaleebu P, Ross A, Morgan D, Yirrell D, Oram J, Rutebemberwa A, Lyagoba F, Hamilton L, Biryahwaho B, Whitworth J: **Relationship between HIV-1 Env subtypes A and D and disease progression in a rural Ugandan cohort**. *Aids* 2001, **15**(3):293-299.

18. Castro H, Pillay D, Cane P, Asboe D, Cambiano V, Phillips A, Dunn DT, Aitken C, Webster D, Chadwick D: **Persistence of HIV-1 transmitted drug resistance mutations**. *Journal of Infectious Diseases* 2013, **208**(9):1459-1463.

19. Uganda Ministry of Health: **Addendum To The Antiretroviral Treatment Guidelines For Uganda**. In*.* Kampala, Uganda; 2013.
