# Supplementary material for: Universal test, treat, and keep: improving ART retention is key in cost-effective HIV control in Uganda
Source: BMC Infect Dis. 2017 May 3;17:322. doi: 10.1186/s12879-017-2420-y (PMC5415795; doi:10.1186/s12879-017-2420-y)
Supplement: Supplementary file 3 — Supporting results. (DOCX 4161 kb) [file 12879_2017_2420_MOESM3_ESM.docx]

# Supporting results

## Combinations of three or more intervention components

Interactions between individual intervention components were identified by comparing the incremental costs and benefits of combined interventions vs single interventions with the costs and benefits of the single interventions. Where interactions between two intervention components were identified, the interacting components were not combined by addition.

There was little interaction between most pairs of individual intervention components in respect to intervention costs and DALYs averted (Figure S3). There were two exceptions to this. The first was when improving ART retention and increasing ART restart rates were combined, the costs and DALYs averted were both lower than when the effects of the two individual components were combined additively. The second is that when removing the threshold for ART initiation was added to the intervention to improve linkage to care, there was slightly greater uncertainty in both the costs and effects of removing the threshold. For this reason, these pairs of interventions were never combined additively. Instead, the simulated combined interventions were used in creating the combinations of three or four intervention components. For instance, when estimating the costs and effects of an intervention combining increased rates of HIV testing, improved retention on ART, and increased ART restart rates, the costs and effects of the simulated ‘improved retention and increased restart rates’ intervention were added to the costs and effects of the simulated increased HIV testing intervention.


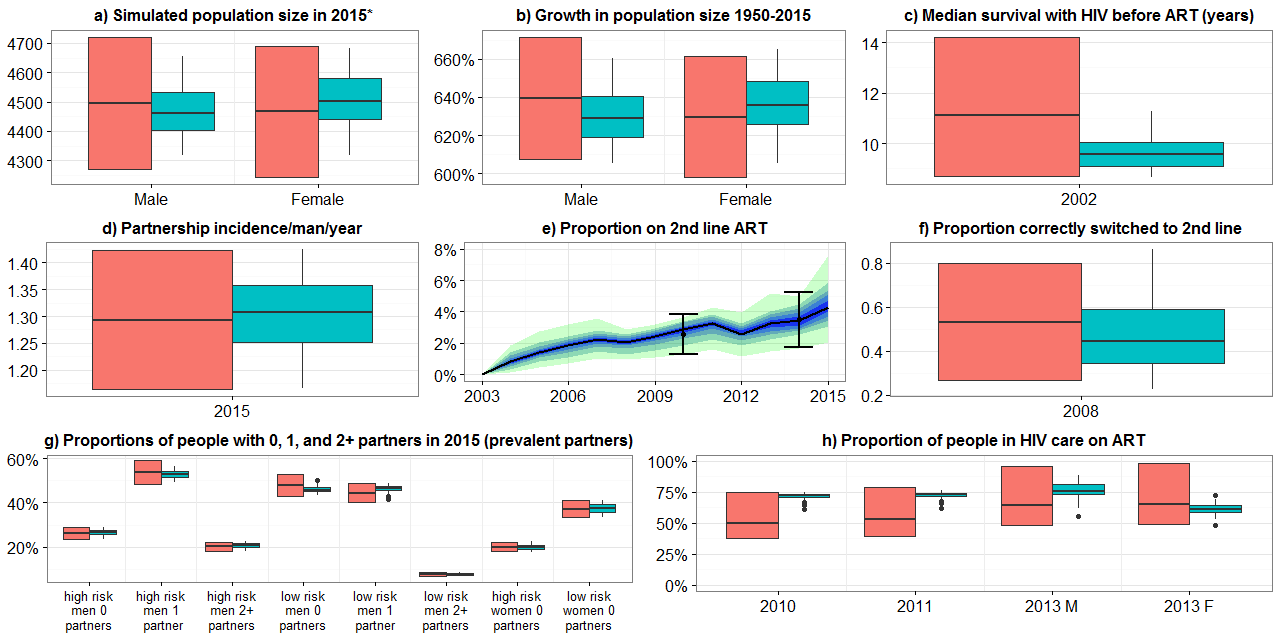


**Figure S1. Model fit to empirical data.** Orange boxes show the empirical data and plausible ranges. Green boxes show the model output. *Simulated population size 1/2000^th^ of the population of Uganda. e) Black dots show the empirical estimates, and the error bars show the plausible ranges for the output values. Black lines show the median model output. Blue/green bands show 10% quantiles of model outputs, from the 100 model fits. The full width of the band shows the range of the model output. f) Proportion of people switched to second line ART in 2008 who were correctly switched due to drug resistance.

**
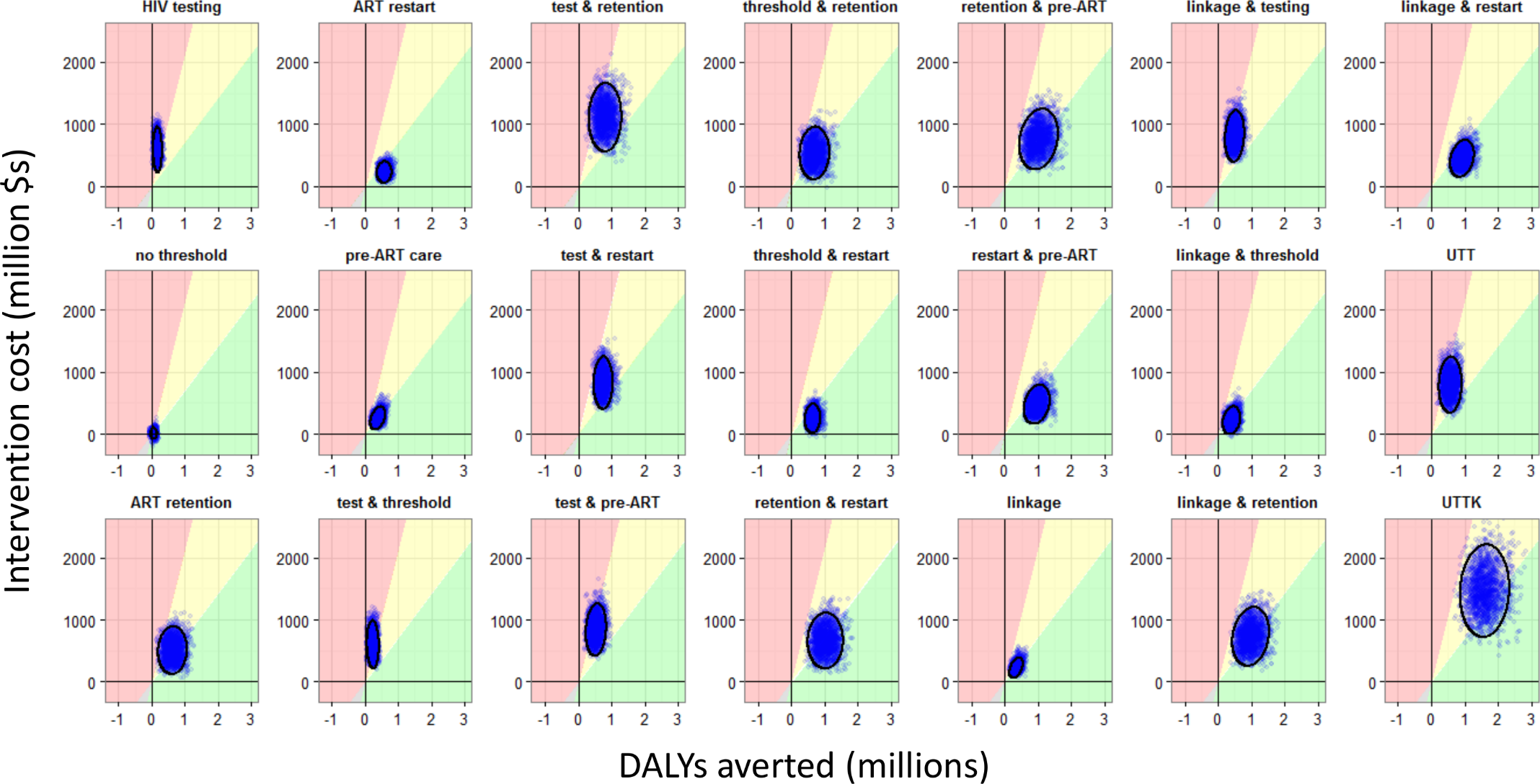
**

**Figure S2. Costs against DALYs averted.**


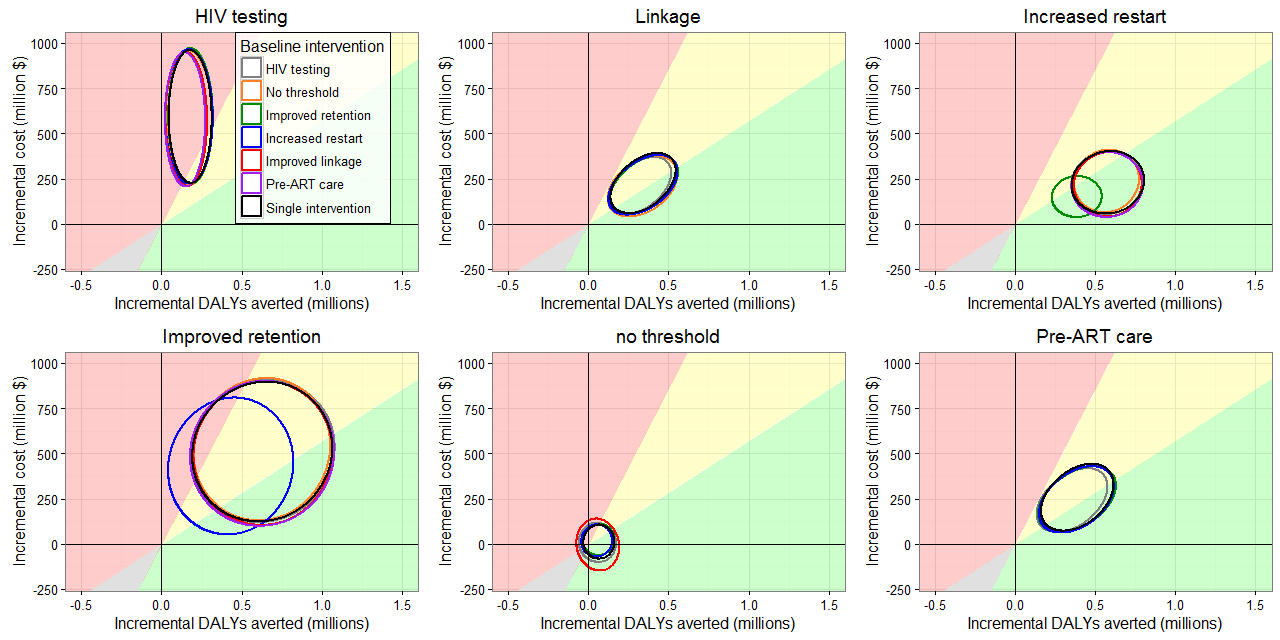


**Figure S3. Effect of changing the baseline scenario on the cost and DALYs averted of intervention components.** Graphs show the costs and effects when the interventions in the graph titles are added to other interventions, listed in the legend. Ovals show approximate 95% plausible ranges. Where the ovals differ greatly from the ovals for the single interventions, it indicates that there is substantial interaction between the two intervention components, and *vice versa*.

**
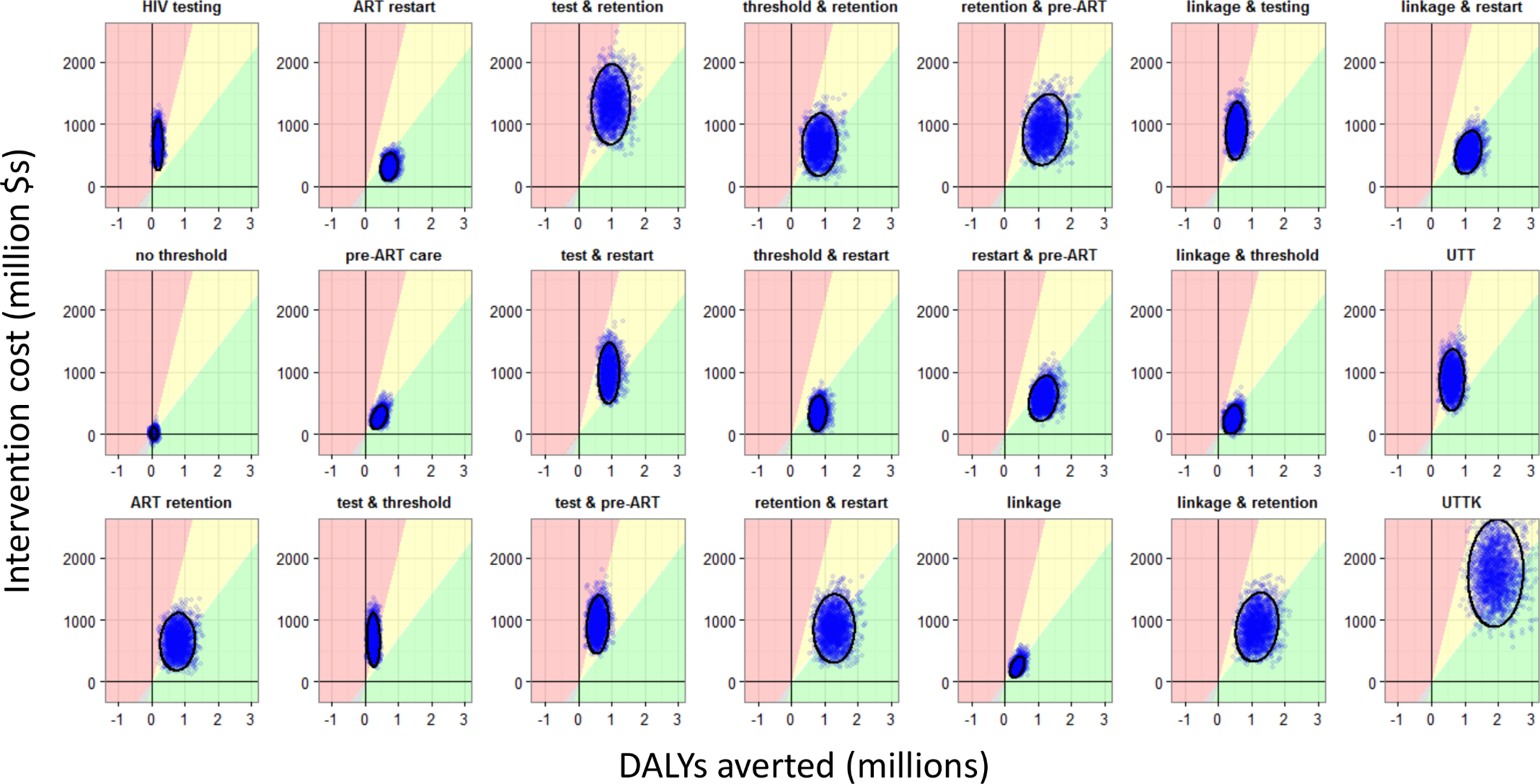
**

**Figure S4. Costs against DALYs averted with a higher maximum age.**

**
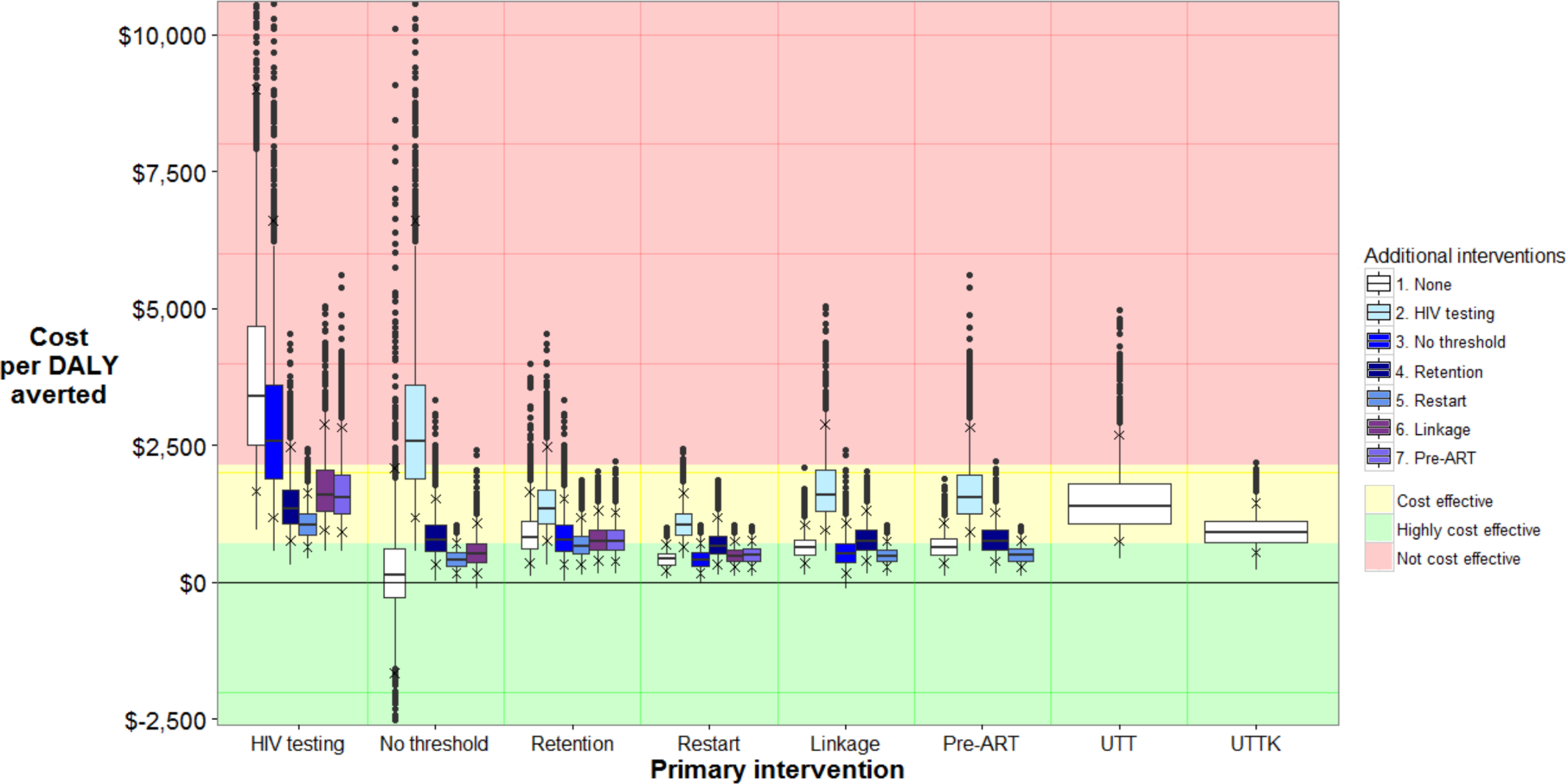
Figure S5. Cost per DALY averted with higher a maximum age.** White boxes show the results for single intervention components, UTT, and UTTK. Shaded boxes show the results for combinations of two intervention components. Boxes show the median and 25-75% quartiles. Crosses show the 90% plausible range. Results for two-component interventions are shown twice, once for each intervention component. Red, yellow, and green bands show areas where intervention are considered not cost-effective (cost >3*Uganda’s per capita GDP per DALY averted, >$1430), cost-effective (cost 1-3* Uganda’s per capita GDP per DALY averted, $715-$1430), and highly cost-effective (cost <1*Uganda’s per capita GDP per DALY averted, <$715) respectively. In this figure, parameter sets are excluded from the results for an intervention if the number of DALYS averted is less than zero. The maximum number of parameters excluded for any intervention is 116/2000 (5.8%).


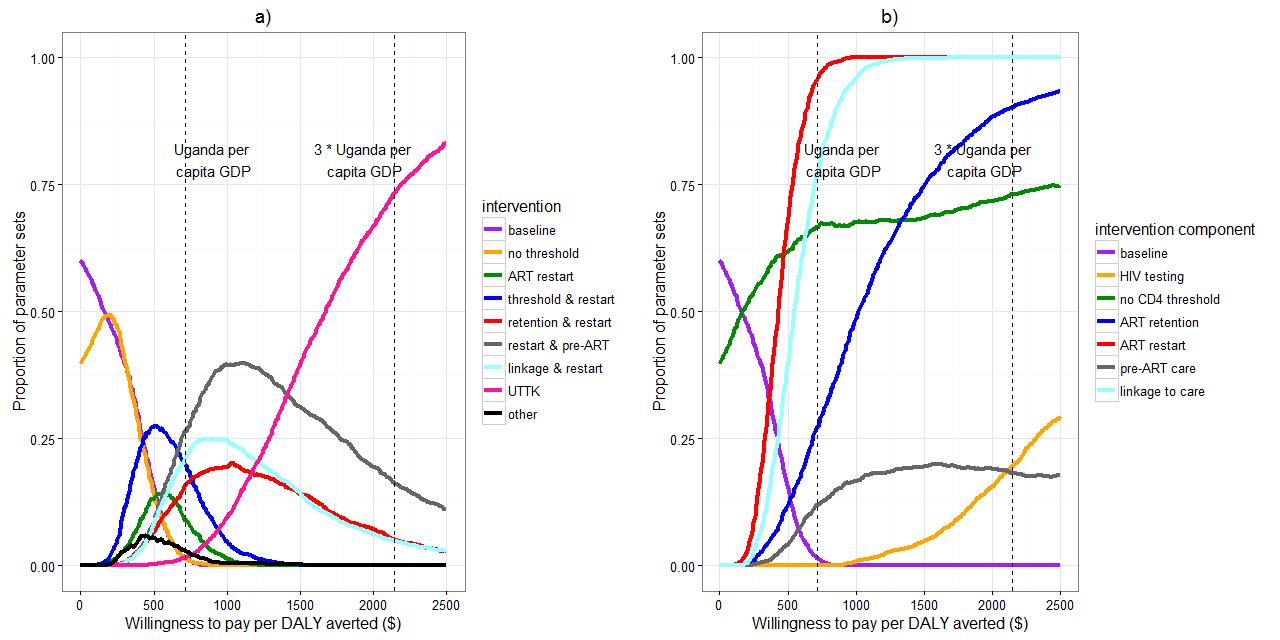


**Figure S6. Cost-effectiveness acceptability curves with higher maximum age.** a) Lines show the proportion of parameter set for which an intervention is the most cost-effective option, for different willingness to pay per DALY averted thresholds. Interventions which are the most cost-effective option in less than 5% of scenarios at all willingness to pay thresholds are combined into the single category ‘other’. b) Lines show the proportion of parameter sets where the most cost-effective intervention includes each individual intervention component, for different willingness to pay per DALY averted thresholds.
