# Supplementary figures and images for: Universal test, treat, and keep: improving ART retention is key in cost-effective HIV control in Uganda
Source: BMC Infect Dis. 2017 May 3;17:322. doi: 10.1186/s12879-017-2420-y (PMC5415795; doi:10.1186/s12879-017-2420-y)

**Figure. Histograms of input paramter values in the 100 model fits**

**
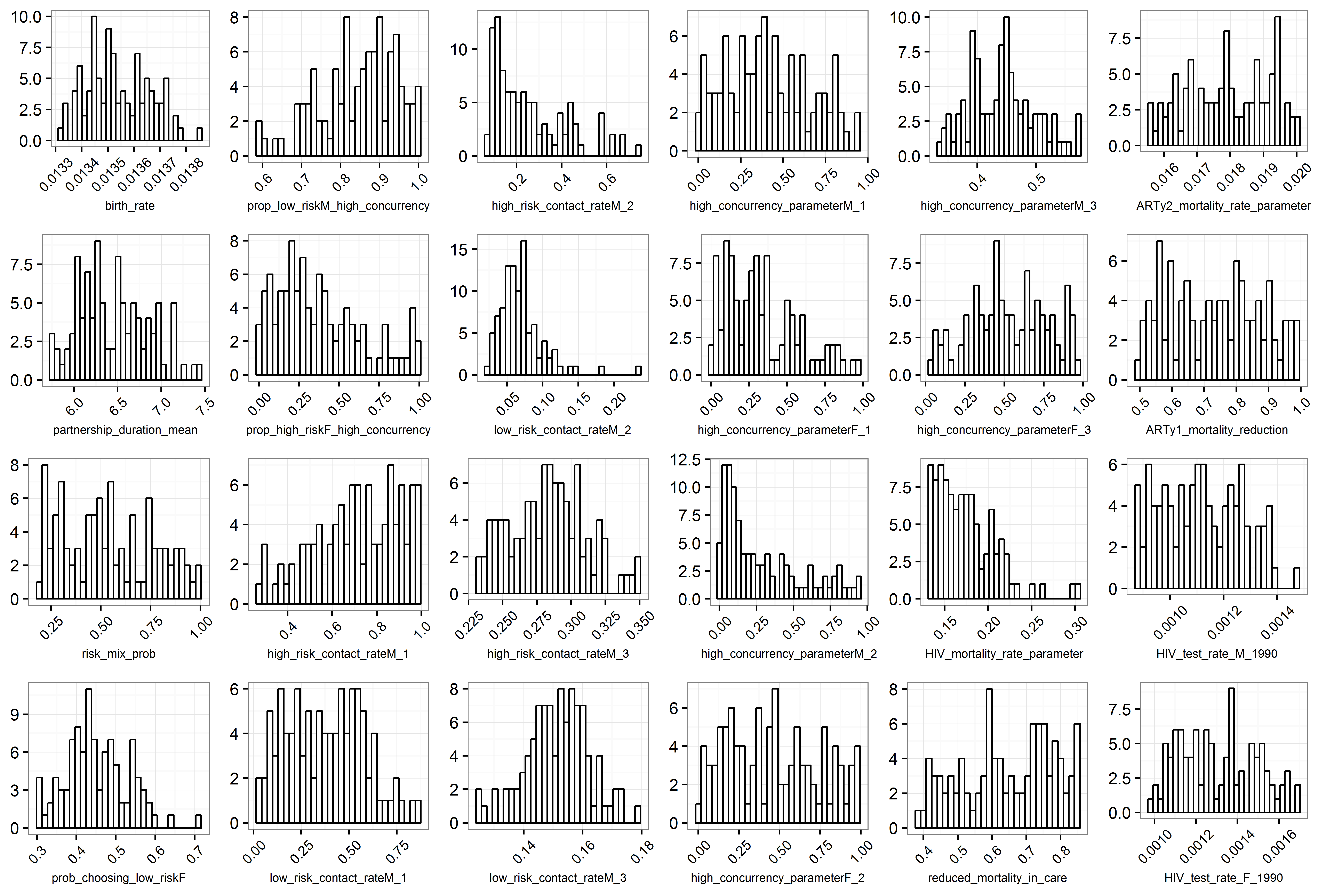

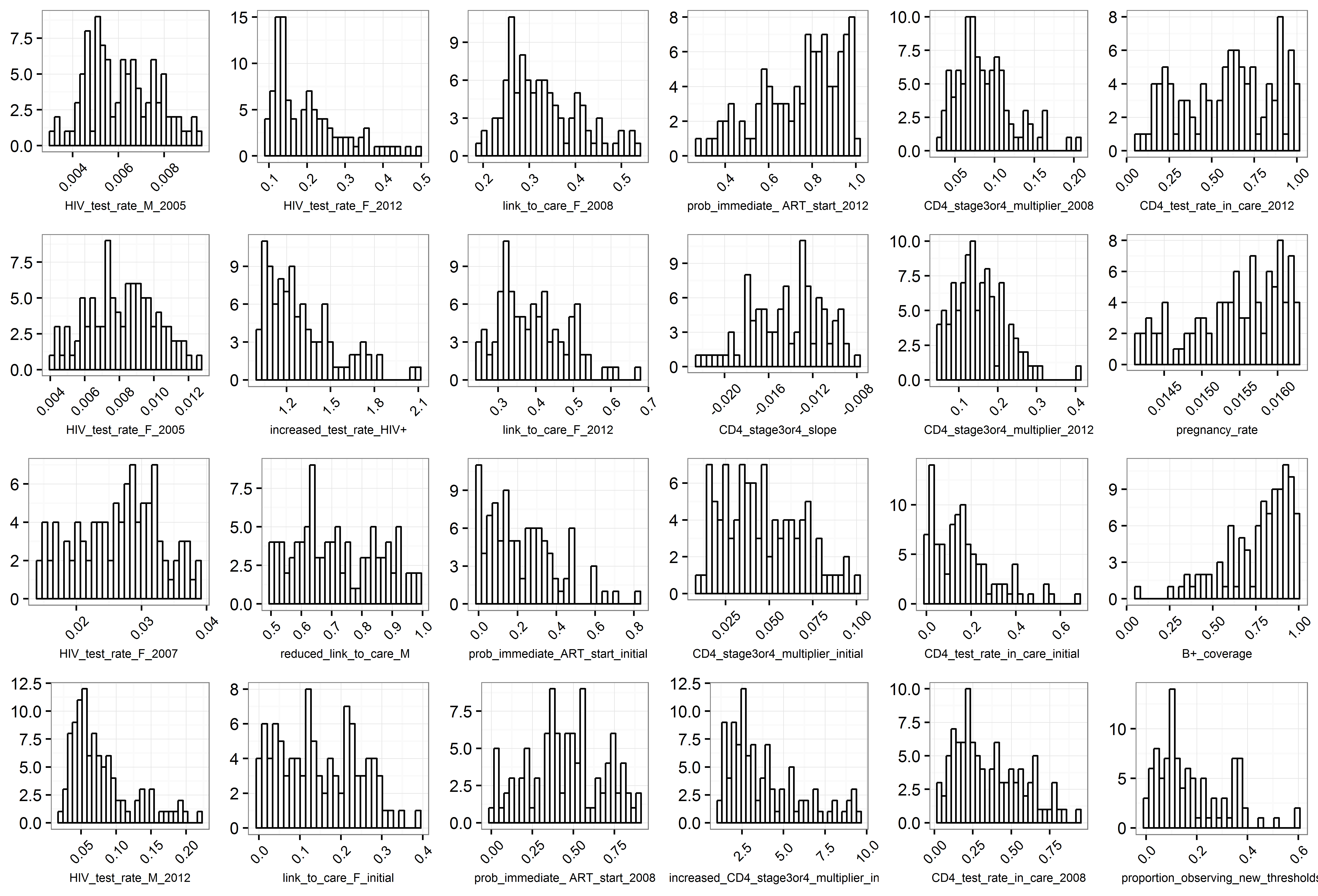

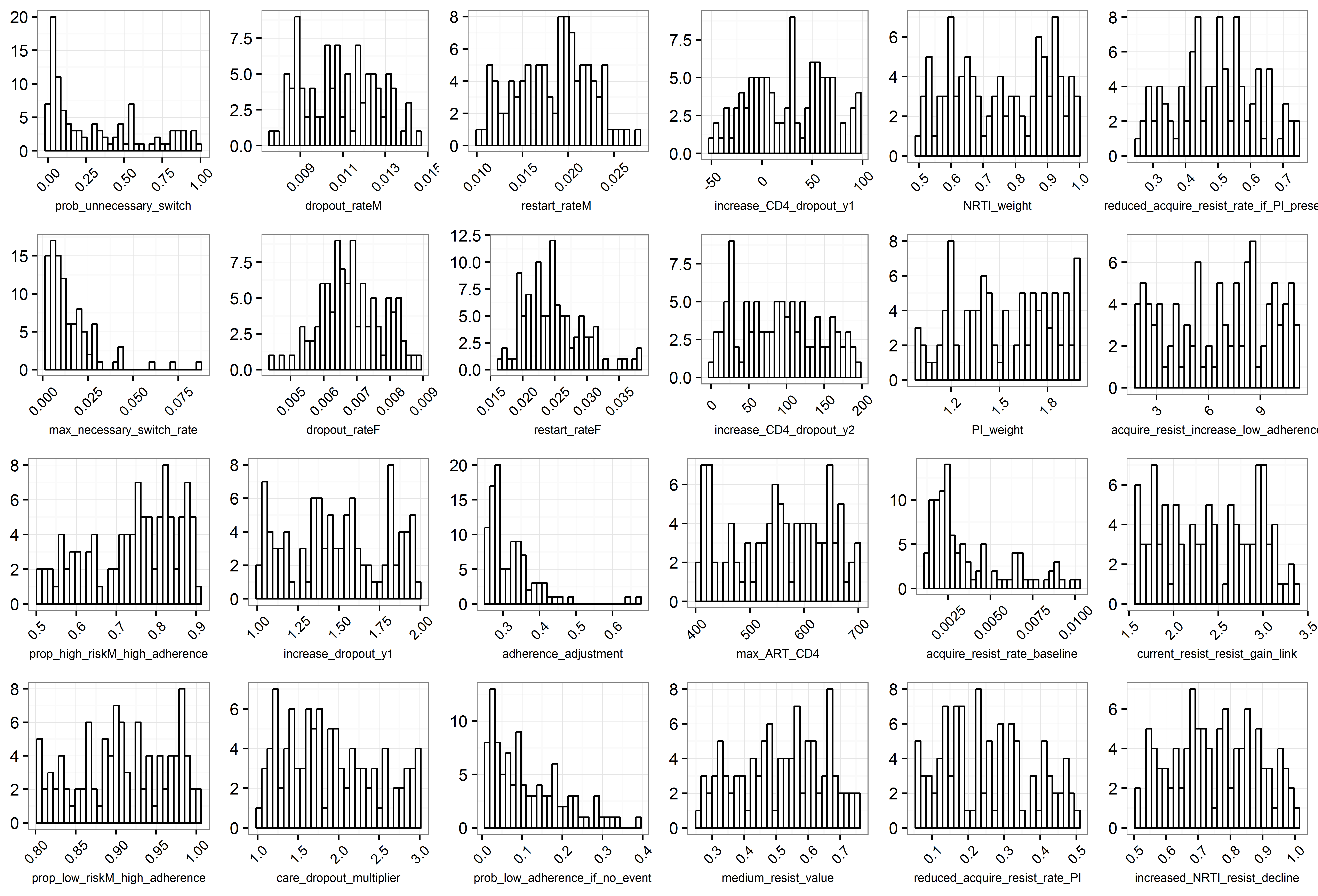

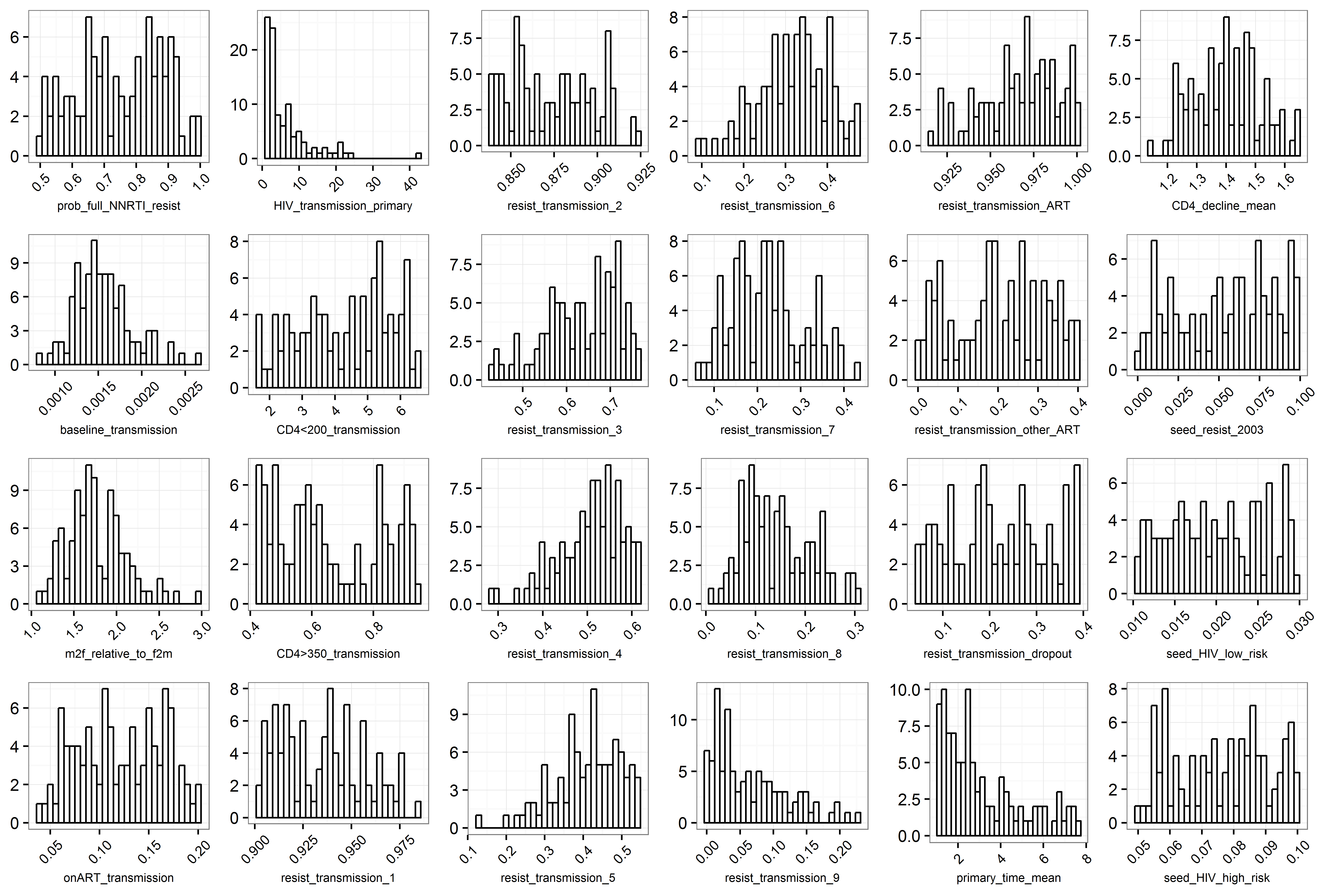
**

Supplement: Supplementary file 4 — Histograms of input parameter values in the 100 model fits. (DOCX 1646 kb) [file 12879_2017_2420_MOESM4_ESM.docx]
